# Acetylation of Lysine 201 Inhibits the DNA-Binding Ability of PhoP to Regulate *Salmonella* Virulence

**DOI:** 10.1371/journal.ppat.1005458

**Published:** 2016-03-04

**Authors:** Jie Ren, Yu Sang, Yongcong Tan, Jing Tao, Jinjing Ni, Shuting Liu, Xia Fan, Wei Zhao, Jie Lu, Wenjuan Wu, Yu-Feng Yao

**Affiliations:** 1 Laboratory of Bacterial Pathogenesis, Department of Microbiology and Immunology, Institutes of Medical Sciences, Shanghai Jiao Tong University School of Medicine, Shanghai, China; 2 Department of Infectious Diseases, Shanghai Ruijin Hospital, Shanghai, China; 3 Department of Laboratory Medicine, Shanghai East Hospital, Tongji University School of Medicine, Shanghai, China; Howard Hughes Medical Institute, Yale University, UNITED STATES

## Abstract

The two-component system PhoP-PhoQ is highly conserved in bacteria and regulates virulence in response to various signals for bacteria within the mammalian host. Here, we demonstrate that PhoP could be acetylated by Pat and deacetylated by deacetylase CobB enzymatically *in vitro* and *in vivo* in *Salmonella* Typhimurium. Specifically, the conserved lysine residue 201(K201) in winged helix–turn–helix motif at C-terminal DNA-binding domain of PhoP could be acetylated, and its acetylation level decreases dramatically when bacteria encounter low magnesium, acid stress or phagocytosis of macrophages. PhoP has a decreased acetylation and increased DNA-binding ability in the deletion mutant of *pat*. However, acetylation of K201 does not counteract PhoP phosphorylation, which is essential for PhoP activity. In addition, acetylation of K201 (mimicked by glutamine substitute) in *S*. Typhimurium causes significantly attenuated intestinal inflammation as well as systemic infection in mouse model, suggesting that deacetylation of PhoP K201 is essential for *Salmonella* pathogenesis. Therefore, we propose that the reversible acetylation of PhoP K201 may ensure *Salmonella* promptly respond to different stresses in host cells. These findings suggest that reversible lysine acetylation in the DNA-binding domain, as a novel regulatory mechanism of gene expression, is involved in bacterial virulence across microorganisms.

## Author Summary

*Salmonella* Typhimurium is a facultative intracellular pathogen capable of survival within host phagocytic cells. The crucial two-component system regulator PhoP-PhoQ is involved in response to different stresses and essential for the virulence of *S*. Typhimurium. In this study, we show that PhoP can be acetylated by the protein acetyltransferase Pat, or deacetylated by the NAD-dependent deacetylase CobB. Acetylation level of the conserved lysine residue 201 (K201) on PhoP varies with environmental factors including low magnesium, acid stress and phagocytosis, which consequently modulates the DNA-binding ability of PhoP. Furthermore, acetylation of PhoP K201 is critical for host inflammation response and bacterial survival in the host. Taken together, these results highlight the fact that *Salmonella* may utilize reversible lysine acetylation to quickly adapt themselves to the hostile environments.

## Introduction

As Gram-negative bacteria, salmonellae can cause enteric diseases in a wide range of animals. *Salmonella* Typhimurium, non-typhoidal *Salmonella* (NTS), is usually used as a model organism to understand bacterial interactions with host cells. *Salmonella* can survive and multiply inside several mammalian cell types, including epithelial cells and macrophages [1, 2]. However, the intracellular *Salmonella* within either epithelial cells or macrophages could escape from neutrophil-mediated killing, which is critical for pathogenesis. *Salmonella* has evolved a plethora of regulatory circuits that facilitate them to adapt to different environments. One important and extensively studied example is the family of two-component signal transduction systems, which are typically comprised of a sensor kinase and a response regulator. PhoP-PhoQ is one such two-component system that has been identified in several bacteria, including *Salmonella* [3], *Yersinia pestis* [4], and *Pseudomonas aeruginosa* [5].

The first evidence of a relationship between PhoP and virulence was provided by a genetic screen in *Salmonella* that *phoP* mutant had survival defect in macrophages [6]. When PhoQ senses the environmental signals such as low concentration of Mg^2+^ [7] or acidic pH produced by neutrophils and macrophages [8–10], it is activated by autophosphorylation. Subsequently, the phosphoryl of PhoQ is transferred to the conserved aspartyl of PhoP, and then the phosphorylated PhoP activates the transcription of *phoP* itself and PhoP-regulated genes. Therefore, the pivotal mission of PhoP for pathogen virulence is mainly attributed to its central role to survival in host [4].

N^ε^-lysine acetylation, as a key post-translational modification, influences protein conformation and/or charge, thus altering DNA-binding affinity, enzymatic activity, protein stability, and protein-protein interactions [11]. In eukaryotes, it is well known that lysine acetylation functions as a critical regulatory mechanism controlling vital cellular processes [12]. Lysine acetylation is also a frequently occurring posttranslational modification in bacteria, however, little is known about its underlying regulatory mechanism [13, 14]. Recent proteomic studies in *E*. *coli* [15–18], *S*. Typhimurium [19], *Bacillus subtilis* [20] and *Mycobacterium tuberculosis* [21] identified several acetylated transcription factors, suggesting that acetylation may regulate gene transcription in bacteria. Furthermore, there are several supporting experimental evidences for gene transcription regulated by acetylation, such as α-CTD of RNA polymerase [22, 23], transcriptional regulator RcsB [24, 25].

Two distinct mechanisms have been revealed in bacteria to regulate the protein acetylation. The first one is enzymatic, whereby the Gcn5-like acetyltransferase Pat/YfiQ transfers the acetyl group from acetyl-CoA (Ac-CoA) to a deprotonated lysine. The second one is non-enzymatic, whereby acetyl phosphate (AcP) serves as the acetyl donor to a deprotonated lysine [13, 14]. Some Pat/YfiQ & Ac-CoA dependent acetyllysines and some AcP-dependent acetyllysines are deacetylated by the nicotinamide adenine dinucleotide (NAD)-dependent deacetylase CobB [16, 18, 26–28]. Currently, it is considered that non-enzymatic acetylation with AcP is more global and less specific than enzyme catalytic acetylation in bacteria [16, 18].

Here we demonstrate that the crucial two-component system regulator PhoP can be enzymatically acetylated by Pat and deacetylated by CobB in *S*. Typhimurium. Acetylation of lysine residue located in the DNA-binding motif inhibits DNA-binding ability of PhoP, and further alters the transcription of *phoP* and PhoP-regulated genes. This enzymatic acetylation of PhoP by Pat is critical to log-phase acid tolerance response (ATR), replication in macrophage, cell inflammation response and virulence to host in *S*. Typhimurium. These findings confirm that reversible N^ε^-Lys acetylation of transcription factors is a conserved regulatory mode of gene expression shared by both eukaryotes and bacteria.

## Results

### PhoP is a substrate of CobB and Pat

**Fig 1:**
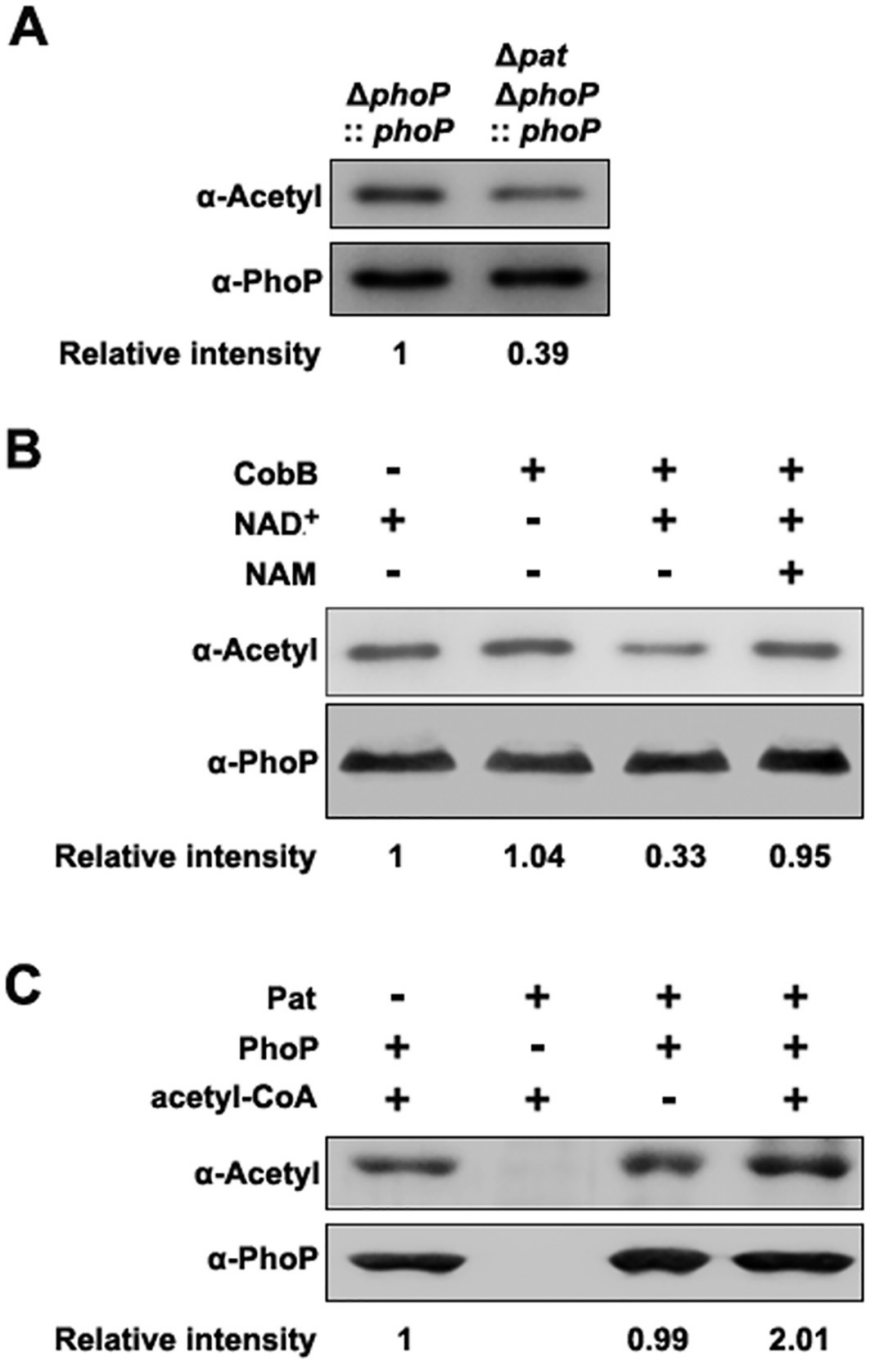
PhoP can be acetylated and deacetylated *in vitro* and *in vivo*. (A) The acetylation levels of PhoP in the wild type strain and *pat* deletion mutant. 6×His-tagged PhoP was expressed using pCDSS-*phoP* in the *pat* deletion mutant or the wild type *S*. Typhimurium. Acetylation levels of the purified PhoP proteins were detected with the pan anti-acetyllysine antibody (α-Acetyl), and the anti-PhoP antibody was used as a loading control. Western blots were independently repeated at least three times. (B) CobB deacetylates PhoP *in vitro*. PhoP (0.2 μg/μl) was purified and incubated with or without CobB (0.1 μg/μl), NAM (10 mM), NAD^+^ (1 mM). The acetylation levels were determined by Western blot. Western blots are representative of at least three independent replicates. (C) Pat acetylates PhoP *in vitro*. PhoP (0.2 μg/μl) was purified from the wild type strain and incubated with or without Pat (0.2 μg/μl) and Ac-CoA (0.2 mM). The acetylation levels were determined by Western blot, and Western blots are representative of at least three independent replicates.

Control of protein function by reversible N^ε^-Lys acetylation is conserved in both eukaryotes and bacteria [24, 29].Proteomic analysis showed that PhoP was acetylated [21], therefore, we want to confirm the observation and determine the mechanism(s) by which PhoP becomes acetylated. To examine whether PhoP can be acetylated in *S*. Typhimurium, we expressed and purified 6×His-tagged PhoP proteins from the wild type strain or *pat* deletion mutant of *S*. Typhimurium. Western blot showed that PhoP proteins from both strains were acetylated, but the acetylation level of PhoP from the *pat* deletion mutant was significantly reduced (approximately 60%) compared with that of PhoP from the wild type strain (Fig 1A). The residual acetylation of PhoP in *pat* deletion mutant suggests there may exist Pat-independent acetylation.

Next, a bacterial two-hybrid system was employed to detect the interactions between PhoP and acetyltransferase Pat or deacetylase CobB. As shown in S1 Fig, both the co-transformants of PhoP/Pat and PhoP/CobB grew well on the screening medium, indicating that PhoP interacts with Pat or CobB. Then, to determine whether PhoP is a substrate of NAD-dependent deacetylase CobB, we purified PhoP from the wild type strain (high acetylation level) and incubated it with CobB at the presence of NAD^+^. The result showed that purified PhoP was acetylated, as shown in Fig 1A. PhoP was a substrate of CobB, with up to 67% removal of the acetyl moiety from PhoP, but not all within 2 h (Fig 1B). The residual acetylation of PhoP may be a CobB-insensitive fraction after CobB treatment. To test whether PhoP is a substrate of Pat, PhoP from the *pat* deletion mutant (low acetylation level) was incubated with Pat at the presence of Ac-CoA. Western blot showed that PhoP can be acetylated by Pat *in vitro*, and the acetylation level increased 110% after treatment (Fig 1C). It indicates that the purified PhoP is acetylated, but not fully, because it can be further acetylated by Pat.

### Acetylation inhibits the transcription of *phoP* and PhoP-regulated genes

**Fig 2:**
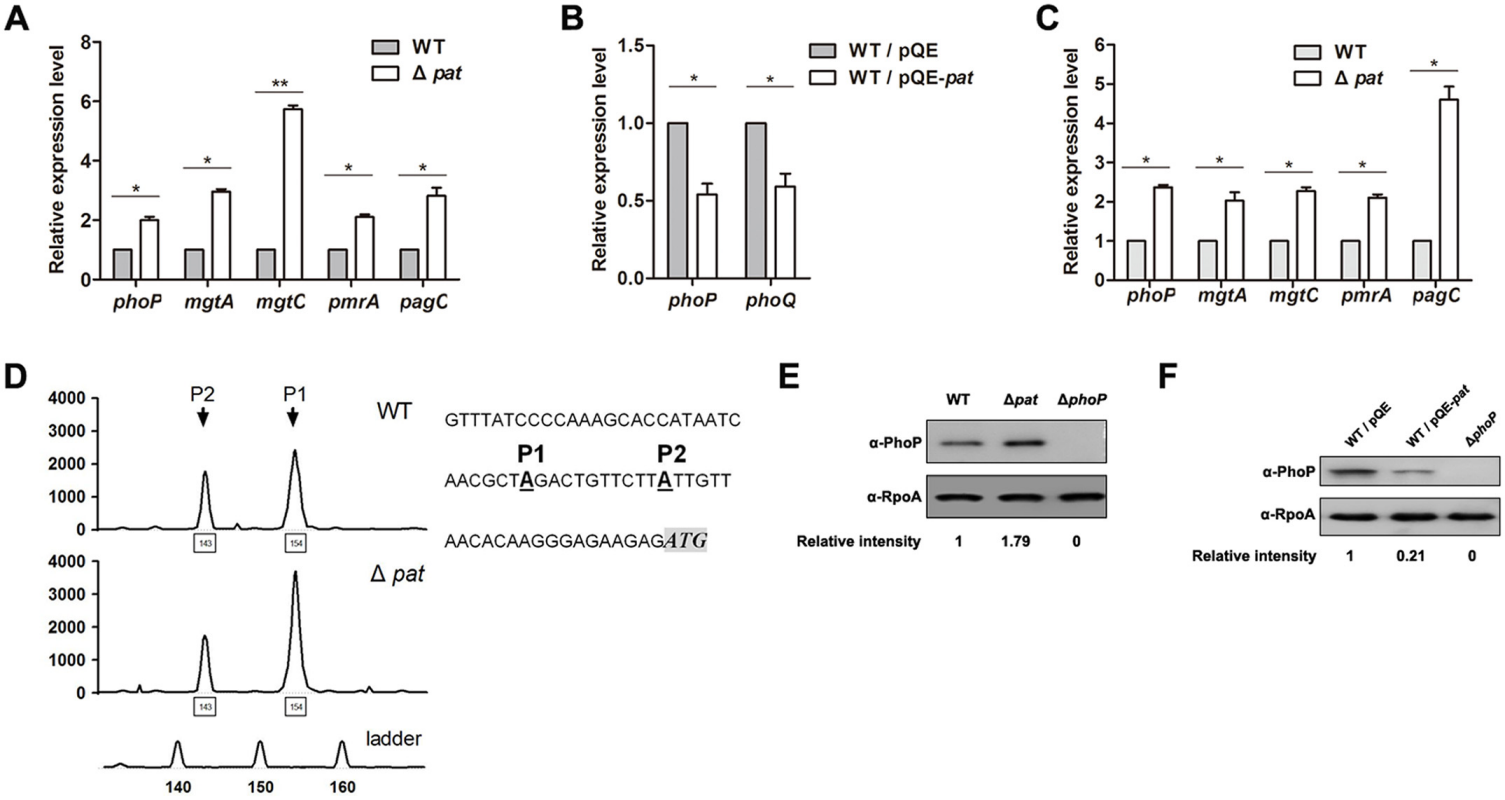
Acetylation is involved in the autoregulation of *phoP*. (A) The transcriptional levels of candidate genes in the wild type strain and *pat* deletion mutant. When bacteria grew to OD_600_~0.4, cells were harvested to isolate total RNA. The transcriptional levels were determined by qPCR with the methods of 2^−ΔΔCt^. The expression of tested gene was normalized to that of 16S rRNA. The relative expression of *phoP* in the *pat* deletion mutant was compared to that in wild type strain (expression level set as 1). *, *P*<0.05; **, *P*<0.01, Student's t test. (B) Gene expression of *phoP* and *phoQ* with overexpression of *pat*. The *pat* was overexpressed using recombinant plasmid pQE80-*pat*, vector pQE80 was transformed into the wild type strain as a control. (C) The transcription levels of *phoP* and PhoP-regulated genes of *S*. Typhimurium in macrophage cells. RAW264.7 cells infected by the wild type strain or *pat* deletion mutant were collected at 24 h post-infection. Bacteria were harvested to isolate total RNA. The transcriptional level was determined by qPCR with the methods of 2^−ΔΔCt^. The relative expression of tested gene was normalized to that of 16S rRNA. *, *P*<0.05; **, *P*<0.01, Student's t test. (D) Primer extension of *phoP* from the wild type strain and *pat* deletion mutant. The graph showed peak fluorescence of the 5’-FAM labeled RT-PCR products of *phoP*. Numbers below each peak indicated the length of the product and the peaks on the bottom line showed the location of size standards used to estimate the length of the transcripts. The location of P1and P2 were shown in the *phoP* promoter sequence. (E) The protein levels of PhoP in the wild type and *pat* deletion mutant. When bacteria grew to OD_600_~0.4, cells were harvested and washed twice with PBS. Sample was mixed with 5 × sample buffer, and boiled for 5 min. PhoP levels were determined by the anti-PhoP antibody, and the anti-RpoA antibody was used as a loading control. Western blots were independently repeated at least three times. (F) The protein level of PhoP with *pat* overexpression. Overexpression of *pat* was achieved by using recombinant plasmid pQE80-*pat*, vector pQE80 was transformed into the wild type strain as a control. Sample from *phoP* deletion mutant was used as a negative control. Western blots are representative of at least three independent replicates.

As a major transcription factor, PhoP regulates the expression of ~5% of genes in *S*. Typhimurium genome, including itself, and the positive feedback boosts the level of PhoP to extend the response range of the PhoP-PhoQ circuit under conditions that strongly activate PhoQ [30]. To determine whether acetylation affects the activity of PhoP as a transcription factor, several known PhoP-regulated genes including *mgtA*, *mgtC*, *pmrA* and *pagC* were selected to evaluate the role of Pat in the regulation of PhoP activity. The quantitative real-time PCR (qPCR) result showed that *phoP* and PhoP-regulated genes were all up-regulated in the *pat* deletion mutant compared with those in the wild type strain (Fig 2A), while the transcriptional level of *phoP* did not change in the *cobB* deletion mutant (S2 Fig). Moreover, overexpression of *pat* decreased the transcription levels of *phoP* and *phoQ* (Fig 2B). We next ask whether deletion of *pat* increases the transcription of *phoP* and PhoP-regulated genes *in vivo*. Then, we used the wild type strain and *pat* deletion mutant to infect mouse macrophage-like RAW264.7 cells, and isolated bacterial RNA from infected macrophage cells. The qPCR result showed that the mRNA levels of *phoP* and PhoP-regulated genes in the *pat* deletion mutant were also elevated significantly compared with the wild type strain in macrophage cells (Fig 2C).

It has been shown that *phoP* has two promoters, the constitutive P2 promoter and autoregulated P1 promoter [7]. To determine whether the observed effects of *pat* deletion on *phoP* transcription are due to the changes in transcription initiated from the autoregulated P1 promoter, the primer extension combined with sequencing assay was performed. The result showed that activity of P1 promoter of *phoP* increased dramatically in the *pat* deletion mutant, while activity of P2promoter did not change (Fig 2D), suggesting that acetylation regulates the activity of the autoregulated P1 promoter instead of the constitutive P2 promoter. To further confirm that PhoP protein level also responds to acetylation, we examined PhoP expression by Western blot, and showed that it increased in the *pat* deletion mutant (Fig 2E) but decreased after overexpression of *pat* (Fig 2F).

### The lysine 201 (K201) is a key residue for PhoP binding to its promoter region

**Fig 3:**
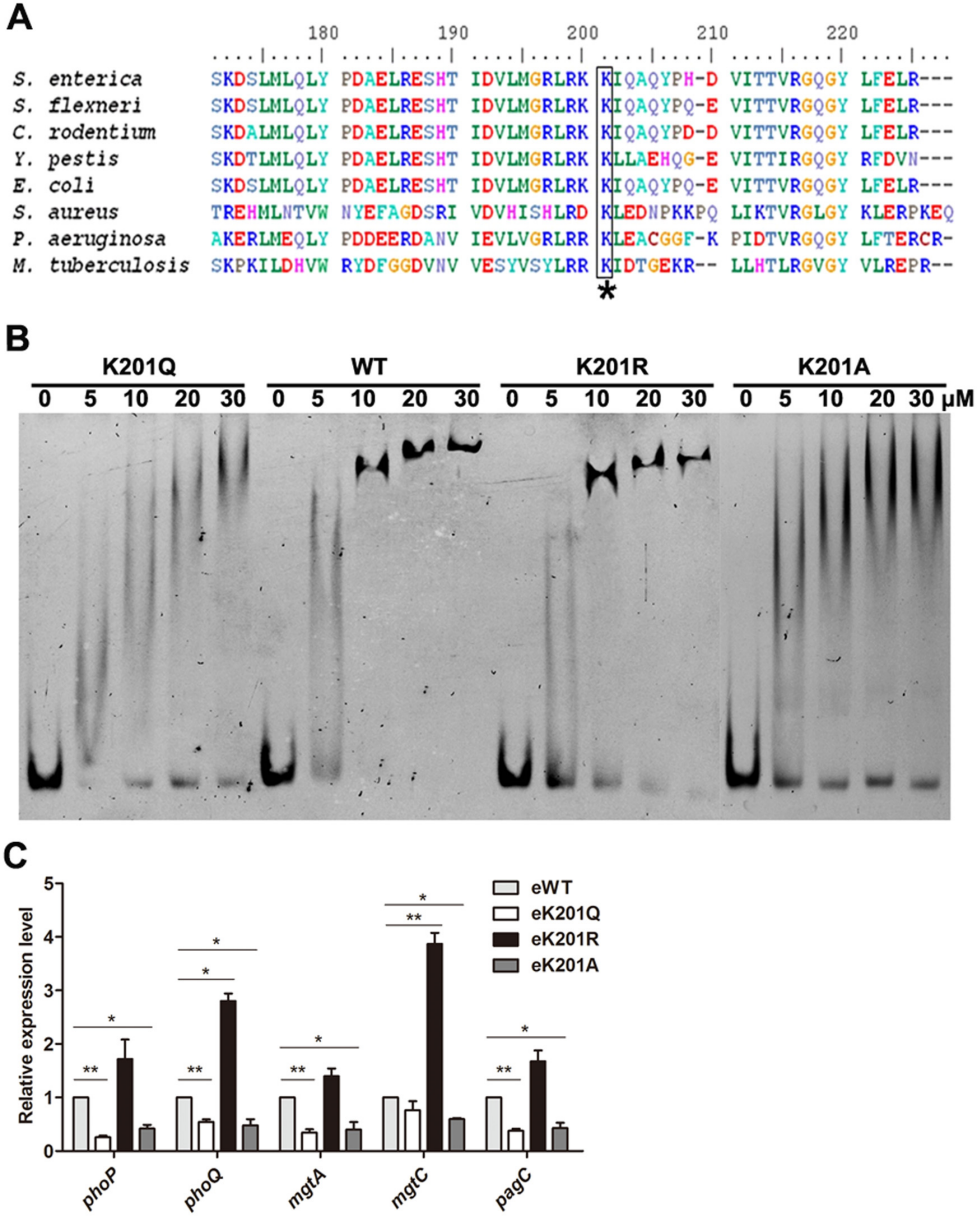
K201 is essential for the activity of PhoP. (A) Conservation analysis of PhoP K201 of *S*. Typhimurium through sequence alignment. Asterisk denotes the conserved lysine residues, and the result was analyzed by BioEdit 7.0. (B) DNA-binding abilities of PhoP and derivatives by EMSA. EMSA was used to test the binding of the indicated concentrations of PhoP (lanes 2 to 5, 7 to 10, 12 to 15, 17–20) to 6’-FAM-labeled *phoP* promoter. Lane 1, 6, 11 and 16 represents the labeled DNA alone. From left to right panel: PhoP K201Q, the wild type PhoP, PhoP K201R and PhoP K201A. EMSA result is representative of at least three independent replicates. (C) The transcription levels of candidate genes in chromosome *phoP* mutants. Bacteria were grown to OD_600_~0.4, and harvested to isolate total RNA. The transcriptional level was determined by qPCR with the methods of 2^−ΔΔCt^. The expression of the tested gene was normalized to that of 16S rRNA and compared to eWT. *, *P*<0.05; **, *P*<0.01, ANOVA analysis.

To identify which lysine residues of PhoP were acetylated, we subjected PhoP gel bands for mass spectrometry analysis. The protein was purified from the *phoP* deletion mutant harboring pCDSS-*phoP* (Δ*phoP*::*phoP*). Three acetylated lysine residues were detected in PhoP, including K18, K150 and K201.Interestingly, K201 was detected more frequently than the other sites. The mass spectrometry of K201 acetylation (K201Ac) was shown in S3 Fig. Importantly, K201 is located within the DNA-binding motif of PhoP and highly conserved in bacteria (Fig 3A). Since the homologous lysine residue of PhoP K201 of *S*. Typhimurium was found to be essential for the PhoP activity in *M*. *tuberculosis* [31], we hypothesize that acetylation of K201 may be involved in regulating the PhoP activity, specifically the DNA-binding ability of PhoP, considering the location and positive charge of this lysine residue. To test this hypothesis, K201 was mutated to arginine (R), glutamine (Q) or alanine (A). Whereas the lysine-to-arginine substitution avoids acetylation but keeps positive charge, thus mimicking the non-acetylated form, the lysine-to-glutamine substitution mimics the constitutively acetylated form through neutralization of the positive charge [32], and lysine residue replaced with alanine cannot be acetylated or deacetylated. Since PhoP with a C-terminal His tag keeps a comparable promoter binding ability as the natural from of PhoP [33], we constructed the following expression plasmids of wild-type PhoP or its derivatives K201A (substituted with alanine), K201Q (substituted with glutamine), K201R (substituted with arginine) with 6×His tag sequence at their C termini. The respective PhoP proteins were overexpressed and purified to apparent homogeneity (S4 Fig). Electrophoretic mobility shift assay (EMSA) was performed by incubating the above purified PhoP derivatives with 6’-FAM-labeled*phoP* promoter followed by non-denaturing polyacrylamide gel electrophoresis analysis. EMSA showed that both K201Q and K201A had lower DNA-binding affinity compared to the wild type PhoP, while K201R possessed the similar DNA-binding ability as the wild type PhoP (Fig 3B). Moreover, the K-to-A mutant tests the importance of the lysine side chain, K-to-Q mutant behaved like the K-to-A mutant (Fig 3B), suggesting K-to-Q nullifies the effect of the side chain. Therefore, K201 residue is indispensable for DNA-binding ability of PhoP, and acetylation of K201 is associated with DNA-binding ability of PhoP *in vitro*.

To examine whether K201 is involved in the regulation of PhoP activity *in vivo*, we constructed chromosome K201Q, K201R, and K201A mutations individually in *S*. Typhimurium, denoted as eK201Q (engineered K201Q), eK201R (engineered K201R), and eK201A (engineered K201A), respectively. In addition, the similar genetic manipulation was performed with the wild type *phoP* and resulted in eWT (engineered WT) as a control. Growth curves and qPCR assays showed that the above genetic manipulations did not affect bacterial growth and transcription of *phoP* or *phoQ* in eWT strain (S5A and S5B Fig). The qPCR assay demonstrated that both K201Q and K201A mutations led to significant transcriptional reduction of *phoP* and PhoP-regulated genes compared with the wild type strain, while K201R mimicking non-acetylated lysine residue activated the transcription of these genes dramatically (Fig 3C), suggesting acetylation of K201 is involved in regulating the binding of PhoP to its DNA site and thus its ability to regulate its regulon in *S*. Typhimurium *in vivo*.

### Site-specific acetylation at K201 causes a defect in PhoP binding to its promoter

**Fig 4:**
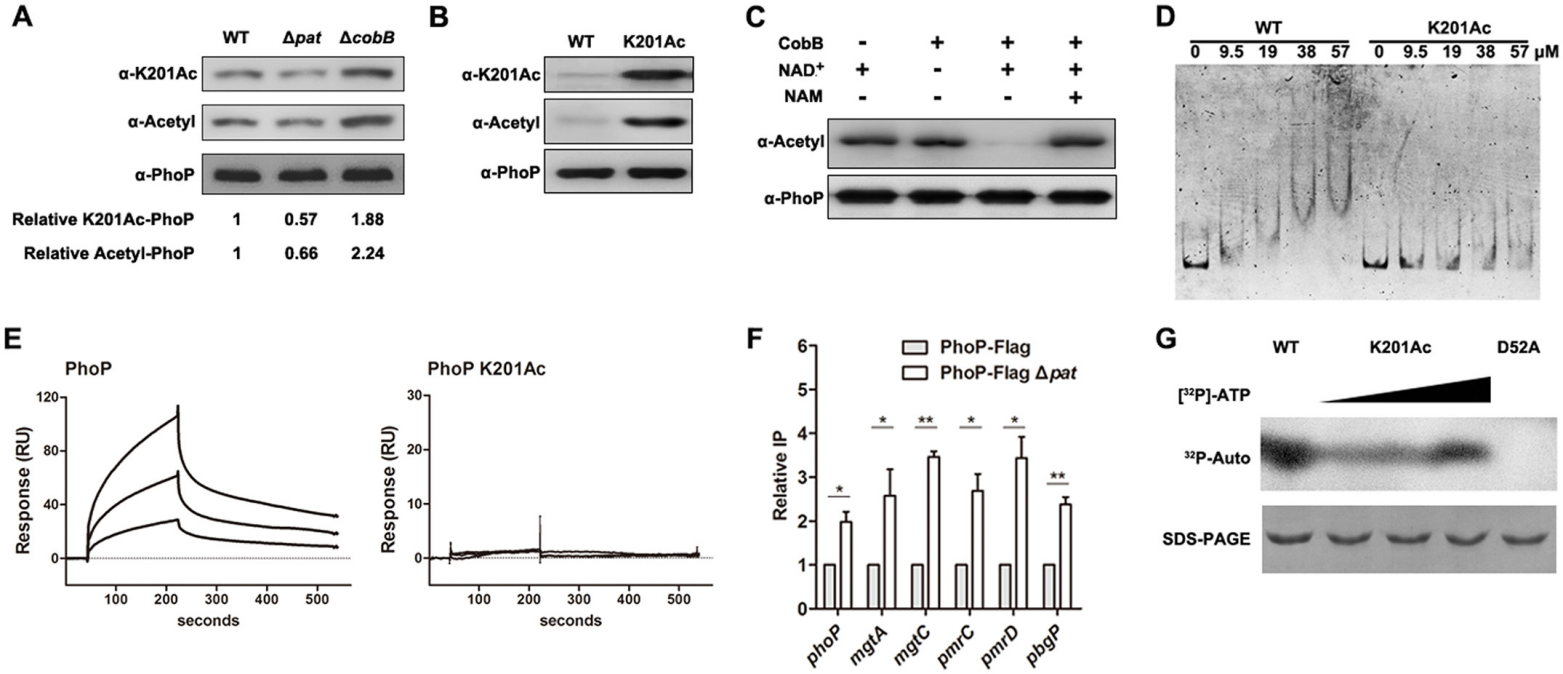
Acetylation of K201 impairs the binding ability of PhoP to its promoter. (A) Acetylation levels of PhoP in different strains. Bacteria were grown to log-phase (OD_600_~0.4) and harvested for IP assay. Flag-fused PhoP proteins were immunoprecipitated by the anti-Flag antibody. The acetylation level of PhoP K201 was determined by the anti-PhoP K201Ac specific antibody, while the acetylation level of PhoP was determined by the pan anti-acetyllysine antibody. Western blots are representative of at least three independent replicates. (B) The acetylation level of PhoP K201Ac. The wild type PhoP and PhoP K201Ac were purified as described in “Materials and Methods” section. The acetylation levels were determined by Western blot using the pan anti-acetyllysine antibody and the anti-PhoP K201Ac specific antibody, respectively. Western blots are representative of two independent replicates. (C) CobB deacetylates PhoP K201Ac *in vitro*. PhoP K201Ac (0.2 μg/μl) was incubated with or without CobB (0.1 μg/μl), NAM (10 mM) and NAD^+^ (1 mM). The acetylation levels were determined by Western blot. Western blots are representative of two independent replicates. (D) DNA-binding abilities of PhoP and PhoP K201Ac. EMSA was used to test the binding of PhoP at indicated concentrations (lanes 2 to 5 and lanes 7 to 10) to 6’-FAM-labeled *phoP* promoter. Lane 1 and lane 6 represent the labeled DNA alone. Left panel: The wild type PhoP was incubated with probes. Right panel: PhoP K201Ac was incubated with probes. EMSA is representative of at least three independent replicates. (E) Binding of PhoP or PhoP K201Ac to the *phoP* promoter by SPR. PhoP was diluted to 1 μM, 0.6 μM, 0.3 μM (up to down) in SPR buffer and injected for 180 s (association phase). This was followed by injection of SPR buffer alone (dissociation phase). Left panel: PhoP, Right panel: PhoP K201Ac. (F) Effect of enzymatic acetylation on *phoP* DNA-binding ability in macrophage. RAW264.7 cells infected by the wild type *S*. Typhimurium strain or *pat* deletion mutant were collected at 24 h post-infection. DNA fragments bound to PhoP were enriched by immunoprecipitation. The relative levels of candidate gene promoters before (Input) and after (Flag-ChIP) IP were determined by qPCR, yielding the ChIP/Input ratio (expressed as %). Error bars represented SD from at least three independent experiments. (G)Acetylation of PhoP K201 does not affect phosphorylation of PhoP. The wild type PhoP (positive control), PhoP K201Ac and PhoP D52A (negative control) were incubated with [^32^P]-AcP, and detected by exposing overnight on a phosphor screen.

To test whether K201 is modified by Pat and CobB *in vivo*, we constructed a chromosome knock-in strain, denoted as PhoP-Flag strain, whose *phoP* gene was fused with Flag-tag sequence at its C terminus in *S*. Typhimurium. The qPCR and growth curves showed that Flag-tag sequence insertion did not affect bacterial growth and transcription of *phoP*-*phoQ* (S5C and S5D Fig). Then, Flag-tagged PhoP proteins were enriched by immunoprecipitation (IP) using the anti-Flag antibody from the *pat* deletion mutant, *cobB* deletion mutant and the wild type *S*. Typhimurium. Consistent with aforesaid result (Fig 1A), we observed PhoP indeed had a lower acetylation level in the *pat* deletion mutant than that in the wild type strain using the pan anti-acetyllysine antibody (Fig 4A). Furthermore, we prepared the anti-PhoP K201Ac specific polyclonal antibody, and its specificity was verified by Western blot (S6 Fig). Western blot using the anti-PhoP K201Ac specific antibody showed that PhoP possessed the lowest acetylation level in the *pat* deletion mutant (0.57-fold of that in the wild type strain), and the highest acetylation level in the *cobB* deletion mutant (1.88-fold of that in the wild type strain) *in vivo* (Fig 4A). It suggests that Pat acetylates and CobB deacetylates K201, but there is also non-Pat-dependent acetylation that is insensitive to CobB.

In order to further study the role of K201 acetylation on DNA-binding of PhoP, the site-specific acetylation incorporated N^ε^-acetyllysine system was used [34]. Firstly, we expressed and purified site-specifically acetylated PhoP (PhoP K201Ac) by genetically encoding the incorporation of N^ε^-acetyllysine in response to the amber stop codon. The *phoP* gene carrying a TAG stop codon at position 201 was co-expressed with an orthogonal N^ε^-acetyllysyl-tRNA synthetase/tRNA_CUA_ pair evolved from methanogenic *Methanosarcina barkeri* when cultured in LB medium supplemented with N^ε^-acetyllysine. This system has been used successfully in several studies to investigate the roles of acetylation on protein functions [29, 35, 36]. PhoP K201Ac was purified to apparent homogeneity (S7 Fig), and the incorporation of acetylated lysine of PhoP K201Ac was verified by Western blot with the pan anti-acetyllysine antibody and the anti-PhoP K201Ac specific antibody, respectively. The acetylation level of PhoP K201Ac increased dramatically compared with the wild type PhoP without site-specific acetylation (Fig 4B). Additionally, when we used CobB to treat PhoP K201Ac, the acetyl groups were removed completely, and this reaction could be inhibited at the presence of CobB inhibitor nicotinamide (NAM) (Fig 4C). It indicates that the acetylation of K201 can be deacetylated by CobB specifically. Combining Fig 4A and 4C, we speculates that K201 could be acetylated by Pat and deacetylated by CobB, but the substantial PhoP acetylation was both Pat-independent and CobB-insensitive. Then, EMSA was used to assess the effect of acetylation at K201 on DNA-binding affinity of PhoP. The PhoP K201Acand the wild type PhoP both containing C-terminal His tag were incubated with 6’-FAM-labeled *phoP* promoter to compare their DNA-binding abilities by EMSA. As shown in Fig 4D, PhoP K201Ac lost its DNA-binding ability compared with non-acetylated PhoP.

To confirm the EMSA result, surface plasmon resonance (SPR) was used to examine, in real-time, the binding of the PhoP and PhoP K201Ac to *phoP* promoter. Biotinylated oligonucleotide duplexes containing the PhoP box of the *phoP* promoter were immobilized on a streptavidin sensor chip, then PhoP and PhoP K201Ac, at concentrations ranging from 0.3 μM to 1 μM, were injected over the DNA surface. Injections of PhoP at 0.6 μM and 1μM over the *phoP* promoter surface resulted in net mass accumulations by 2.3- and 4.0- fold higher than the one with 0.3 μM PhoP injection, respectively. However, injection of PhoP K201Ac even at a concentration of 1 μM did not cause net mass accumulation (less than 5 resonance units) (Fig 4E). This result indicates that acetylation of PhoP K201 abolished the DNA-binding ability of PhoP.

Since the acetylation level of K201 is regulated by Pat, and the acetylation of K201 impairs DNA-binding next we wonder whether Pat could regulate DNA-binding ability by acetylation of PhoP in the host cells. To this end, we used chromatin immunoprecipitation (ChIP) to detect DNA-binding ability of PhoP in the wild type strain and *pat* deletion mutant phagocytized by RAW264.7 cells. As we expected, PhoP could bind more promoter DNA of *phoP* and PhoP-regulated genes in the *pat* deletion mutant in macrophage cells (Fig 4F).

It is well known that phosphorylation is essential for DNA-binding ability of PhoP *in vivo* [37]. To determine whether the effects of acetylation on DNA-binding ability are due to the altered phosphorylation of PhoP, we performed phosphorylation assay *in vitro*. We incubated PhoP K201Ac with the high-energy phosphodonor acetyl phosphate (AcP), which is known to phosphorylate many bacterial response regulator (RRs) including PhoP [38] and has been successfully used as a proxy for phosphorylation of RRs by their cognate kinase [39].The result showed that PhoP was phosphorylated in the presence of [^32^P]-AcP, while the mutant form of PhoP with the D52A (aspartate-to-alanine substitution) amino acid substitution in its predicted phosphorylation site could not be phosphorylated. However, PhoP K201Ac still could be phosphorylated and its phosphorylation signals increased along with the raised concentration of [^32^P]-AcP (Fig 4G). This result demonstrates that the acetylation of K201 did not counteract PhoP phosphorylation.

### Acetylation of PhoP K201 causes an attenuated response to magnesium and acid tolerance response

**Fig 5:**
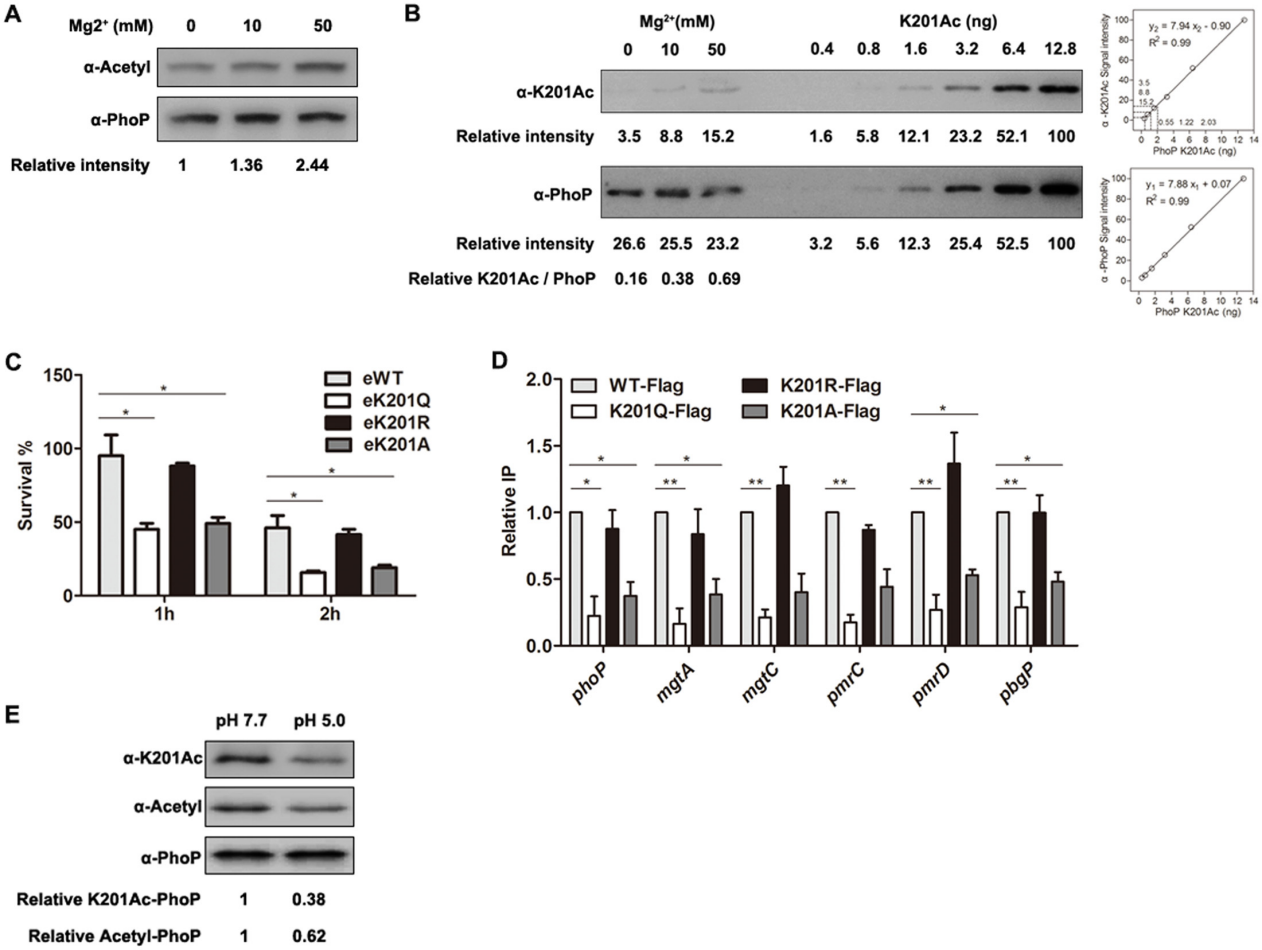
PhoP K201 acetylation is involved in magnesium and acid tolerance response. (A) Acetylation levels of PhoP under different concentrations of magnesium. Bacteria were grown to log-phase in LB supplemented with different amount of magnesium. Flag-fused PhoP proteins were immunoprecipitated by the anti-Flag antibody. The acetylation level of PhoP was determined by the pan anti-acetyllysine antibody (α-Acetyl). Western blots are representative of at least three independent replicates. (B) Acetylation proportion of K201 under different concentrations of magnesium. Flag-fused PhoP proteins were immunoprecipitated by the anti-Flag antibody, whose acetylation fraction of K201 was determined by the anti-PhoP K201Ac specific antibody (α-K201Ac) and the anti-PhoP antibody (α-PhoP) by 2-fold serial dilution of PhoP K201Ac. Set the α-PhoP gray value to “y1”, the α-K201Ac gray value to “y2” and the protein content to “x” to draw the two scatter diagrams. The standard curves were shown as indicated. The gray values of three samples were mapped to the standard curves, so as to get the content of total PhoP (3.37 ng, 3.23 ng and 2.94 ng) and K201Ac (0.55 ng,1.22 ng and 2.03 ng). Finally, the acetylation fraction of each sample was K201Ac/ total PhoP (16%, 38% and 69%). (C) The survival rates of chromosome *phoP* mutants in log-phase ATR. Log-phase *S*. Typhimurium cells were adapted in EG medium at pH 5.8 for 1 h to induce ATR, and then cells were harvested and resuspended in the same volume of EG medium at pH 3.3. After 1 h or 2 h incubation, viable counts were recorded. *, *P*<0.05, ANOVA analysis. (D) Different DNA-binding abilities of K201 mutations in log-phase ATR. Chromosome Flag-tag sequence fused *phoP* mutants were treated by EG medium at pH 5.0 for 1 h, and cells were collected and the DNA fragments bound to PhoP were immunoprecipitated according to the ChIP assay presented in the Materials and Methods section. The relative amounts of candidate gene promoters before (Input) and after (Flag-ChIP) IP were determined by qPCR, yielding the ChIP/Input ratio (expressed as %). Error bars represented SD from at least three independent experiments. *, *P*<0.05; **, *P*<0.01, ANOVA analysis. (E) Acetylation level of PhoPK201 after acid stimulation. After treatment with EG medium at pH 5.0or pH 7.7 for 1 h, cells were harvested for IP assay. Flag-fused PhoP proteins were immunoprecipitated by the anti-Flag antibody. The acetylation level of PhoP K201 was determined by the anti-PhoP K201Ac specific antibody (α-K201Ac), while the acetylation level of PhoP was determined by the pan anti-acetyl lysine antibody (α-Acetyl). Western blots are representative of at least three independent biological replicates.

As we know, *S*. Typhimurium responds to environmental divalent cation limitation, including magnesium and calcium by inducing the transcription of the PhoP-PhoQ regulon [40]. To clarify whether magnesium concentration alters the acetylation level of PhoP *in vivo*, Flag-tagged PhoP proteins were immunoprecipitated by the anti-Flag antibody from bacteria cultured in LB medium supplemented with different amount of magnesium. With the pan anti-acetyllysine antibody, we observed an elevated acetylation level of PhoP along with increase of magnesium concentration (Fig 5A). To further determine whether acetylation levels of PhoP K201also responds to magnesium concentrations, the anti-PhoP K201Acspecific antibody was applied to examine acetylation levels of PhoP K201. As shown in Fig 5B, there was a 4.11-fold increase of acetylation level in PhoP K201 coupled with the increasing concentration of magnesium from 0 mM to 50 mM. It suggests that *Salmonella* may regulate acetylation level of PhoP K201 in response to environmental magnesium concentration. We then quantified the acetylation proportion of PhoP K201 in different concentrations of magnesium. It was accepted that the acetylation level of site-specifically acetylated PhoP K201 was 100% [34]. Western blot showed that the acetylation levels of PhoPK201Ac were positively correlated to PhoP K201Ac protein amounts in the tested protein concentration ranges by 2-fold serial dilutions (Fig 5B).This linear association between the acetylation level of PhoPK201Ac and protein amount makes it applicable to quantify acetylation fraction of PhoPK201 with the anti-PhoP K201Ac specific antibody. Briefly, the band intensity ratio of PhoP detected with anti-PhoP K201Ac against that detected with anti-PhoP could be regarded as the percentage of K201Ac among total PhoP proteins. Thus, we calculated that the fractions of PhoP K201acetylation under treatment with 0 mM, 10 mM and 50 mM magnesium were about 16%, 38% and 69%, respectively. It has been shown that the transcription of *phoP* and PhoP-regulated genes were completely inhibited by 10 mM magnesium [41], which caused about one third of PhoP K201 was acetylated, suggesting low acetylation proportion of PhoP K201 is required for PhoP activity.

The acid tolerance response (ATR) of *S*. Typhimurium is a complex inducible phenomenon in which exposure to slight or moderate low pH causes a stress response to protect the organism from more severe acid challenges. The transient development of acid tolerance was correlated to the temporary induction of a subset of acid shock proteins (ASPs) [42, 43]. Moderate acid pH has been shown to promote the transcription of several PhoP-regulated genes involved in the log-phase ATR of *S*. Typhimurium [10], mainly conferring protection from acid stress. The observation that *phoP* mutant is sensitive to lethal acid treatment and fails to induce the expression of ASPs established the essential role of *phoP* in acid tolerance [10]. To investigate the role of PhoP acetylation in ATR, we challenged the eK201Q, eK201R, eK201A as well as eWT of *S*. Typhimurium with EG medium at pH 5.8 for 1 h, then collected and resuspended cells with same volume of EG medium at pH 3.3. After 1h or 2 h incubation, viable bacteria were enumerated. C.f.u. counting results showed that both eWT and eK201R were more acid-resistant than eK201Q and eK201A after acid exposure. After 2 h of exposure at pH 3.3, the survival of eK201Q mimicking acetylation was less than 25% (Fig 5C), while the survival of eK201R mimicking non-acetylation was about 50%, suggesting that acetylation of PhoP K201 is detrimental for bacterial survival in acid stress. To further determine the impaired ATR was due to variant DNA-binding affinity of PhoP derivatives, ChIP assay was performed to determine DNA-binding abilities of Flag-tagged PhoP and its derivatives (denoted as K201R-Flag, K201Q-Flag and K201A-Flag). qPCR using the DNA samples immunoprecipitated with the anti-Flag antibody showed a similar PhoP-associated DNA enrichment in the wild type PhoP-Flag as well as K201R-Flag. However, PhoP-associated DNA enrichmentinK201Q-Flag and K201A-Flagwere much less compared with that of WT-Flag (Fig 5D).

Next, to determine the acetylation level of PhoP K201 *in vivo* after mild acid treatment, Flag-tagged PhoP proteins were immunoprecipitated with the anti-Flag antibody followed by Western blot using the anti-PhoP K201Ac specific antibody and the pan anti-acetylation antibody. Approximately 60% of acetylation PhoP K201 diminished after stimulation from medium pH 7.7 to medium pH 5.0 (Fig 5E). These results demonstrate that *S*. Typhimurium promotes PhoP binding affinity to PhoP-regulated genes’ promoters in log-phase ATR by downregulating the acetylation level of PhoP K201.

### Acetylation of PhoP K201 impairs bacterial intracellular replication and inflammation response in macrophages

**Fig 6:**
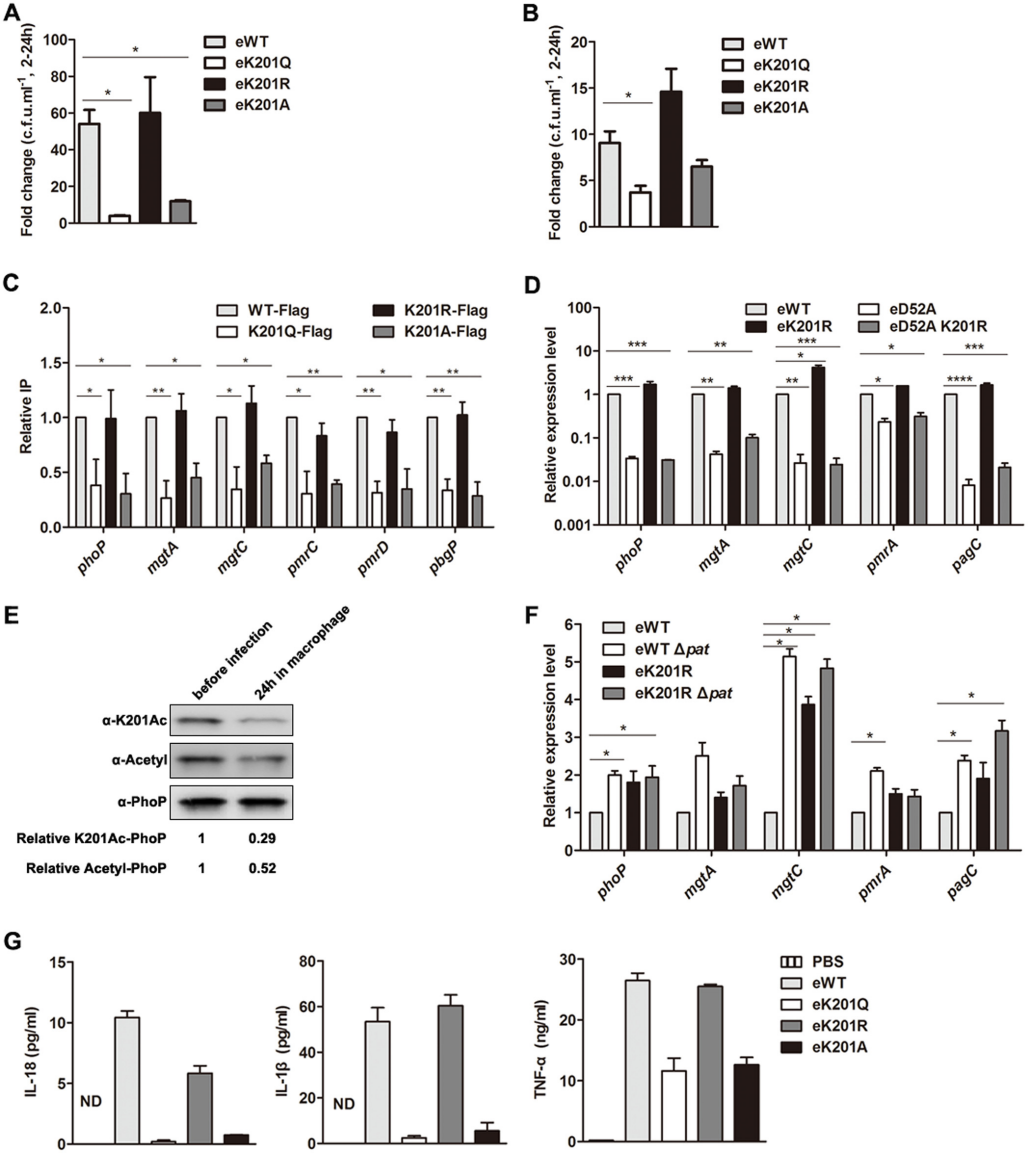
Acetylation of PhoP K201 regulates intracellular replication and inflammation response in macrophages. (A) The replication of bacteria in RAW264.7 cells. Net growth between 2 h and 24 h was calculated from the fold change in c.f.u. /ml recovered at these time points. Error bars represented SD from at least three independent experiments. (B) The replication of bacteria in primary peritoneal macrophages. Net growth between 2 h and 24 h was calculated from the fold change in c.f.u. /ml recovered at these time points. Error bars represented SD from at least three independent experiments. (C) Effect of the mimic acetylated or non-acetylated K201 on its DNA-binding ability in macrophage. RAW264.7 cells infected by chromosome PhoP-Flag mutants were collected at 24 h post-infection. DNA fragments bound to PhoP were enriched by immunoprecipitation. The relative levels of candidate genes’ promoters before (Input) and after (Flag-ChIP) IP were determined by qPCR, yielding the ChIP/Input ratio (expressed as %). Error bars represented SD from at least three independent experiments. (D) The transcription levels of candidate genes in chromosome *phoP* mutants. Bacteria were grown to OD_600_~0.4, and harvested to isolate total RNA. The transcriptional level was determined by qPCR with the methods of 2^−ΔΔCt^. The relative expression of tested gene was normalized to that of 16S rRNA. (E) Acetylation level of PhoP K201 in macrophages. After 24 h treatment, the intracellular bacteria were harvested for IP assay. Flag-fused PhoP proteins were immunoprecipitated by the anti-Flag antibody. The acetylation level of K201 was determined by the anti-PhoP K201Ac specific antibody (α-K201Ac), while the acetylation level of PhoP was determined by the pan anti-acetyllysine antibody (α-Acetyl). Western blots are representative of at least three independent biological replicates. (F) Comparison of the transcription levels of candidate genes between the wild type PhoP strain and PhoP K201R mutant on the *pat* deletion background. Bacteria were grown to OD_600_~0.4, and harvested to isolate total RNA. The transcriptional levels of candidate genes were determined by qPCR with the methods of 2^−ΔΔCt^. (G) Cytokines detection by ELISA. RAW264.7 cells were infected with chromosome PhoP mutants (MOI = 10) for 24 h. Cell culture supernatants were collected to detect the amounts of several cytokines after infection. Error bars represented SD from at least three independent experiments. *, *P*<0.05; **, *P*<0.01, ANOVA analysis.

Earlier studies revealed that the two-component system PhoP-PhoQ is essential for *S*. Typhimurium virulence. The deletion of *phoP* or *phoQ* decreased bacterial survival in macrophages [44]. Subsequently, more studies showed that PhoP governs both *Salmonella* pathogenicity island 1 (SPI-1) [45] and *Salmonella* pathogenicity island 2 (SPI-2) to mediate bacterial virulence [46]. Therefore, we wonder if PhoPK201 acetylation is critical for bacterial survival and proliferation in macrophages. To answer this question, RAW264.7 cells were infected with eWT or eK201 mutants of *S*. Typhimurium with multiplicities of infection of 10. At indicated time points, cells were lysed, and the number of intracellular bacteria was calculated by c.f.u. counting. The result showed that both eWT and eK201R proliferated by an approximately 50-fold in macrophages from 2 h to 24 h post-infection. In contrast, the eK201Q and eK201A exhibited only around 10-fold proliferation (Fig 6A). Additionally, primary peritoneal macrophage cells were used to confirm the observation in RAW264.7 cells. As expected, eWT and eK201R multiplied more efficiently than eK201Q or eK201A in primary peritoneal macrophage cells (Fig 6B). These results demonstrate that eK201Q mimicking N^ε^-Lys acetylation attenuated bacterial proliferation in macrophages compared with eWT and eK201R, irrespective of macrophage types.

Next, to clarify whether the attenuated intracellular replication of eK201Q and eK201A is due to defect in the DNA-binding affinity of PhoP *in vivo*, ChIP assay was performed to detect DNA bound to PhoP of bacteria in macrophage cells. The enriched candidate DNA samples were quantified by qPCR. The ChIP assay showed that K201Q-Flag and K201A-Flag possessed similar DNA-binding abilities, which were about half of these of PhoP-Flag or K201R-Flag in macrophage (Fig 6C), suggesting that acetylation of K201 inhibits the DNA-binding of PhoP during infection. In addition, we introduced the D52A mutation into the eK201R background, and qPCR showed that the transcriptional levels of PhoP-regulated genes decreased significantly ineD52A compared with those of eWT. The transcriptional levels of the tested genes in eD52A/K201R double point mutant were comparable to those in eD52A (Fig 6D). This result not only confirmed the previous idea that phosphorylation is required for PhoP function *in vivo*, but also suggested that phosphorylation is indispensible for acetylation-regulated PhoP activity.

Since mutation mimicking acetylation showed that acetylation of K201 inhibited PhoP DNA-binding ability *in vivo*, we speculate that acetylation level of PhoP K201 might be decreased in macrophage. To determine the acetylation level of PhoP K201 in macrophage, Flag-tagged PhoP proteins were immunoprecipitated after 24h infection followed by Western blot analysis using the anti-PhoP K201Ac specific antibody. The result showed a dramatic decrease of acetylation level of PhoP K201 in intracellular bacteria compared with bacteria cultured in LB medium (Fig 6E). In addition, qPCR detected similar transcription levels of the PhoP-regulated genes between eWT and eK201R on the *pat* deletion background (Fig 6F), suggesting that K201 is the major acetylation site of PhoP regulated by Pat. Taken together, these results imply that *S*. Typhimurium promptly activates PhoP by downregulating the acetylation level of K201 for better survival and proliferation in macrophage cells.

It is well known that the invasion of *Salmonella* activates caspase-1 and triggers the release of the pro-inflammatory cytokines interleukin (IL)-18 and IL-1β by macrophages [47, 48]. Additionally, TNF-α, as a main pro-inflammatory cytokine with antibacterial activity against *Salmonella* can be produced by macrophages [49]. To explore whether acetylation of PhoP affects the inflammation activation in macrophage, cell supernatants were collected after *S*. Typhimurium infection, and the levels of several cytokines were detected by ELISA. As shown in Fig 6G, infection of eWT or eK201R triggered macrophages to secrete cytokines including IL-18, IL-1β and TNF-α. However, secretion of the above cytokines was much less in eK201Q- or eK201A-infected cells. Therefore, the result suggests that acetylation of PhoP K201 attenuates inflammatory response in macrophage cells.

### Acetylation of PhoP K201 is hypovirulent in mouse model

**Fig 7:**
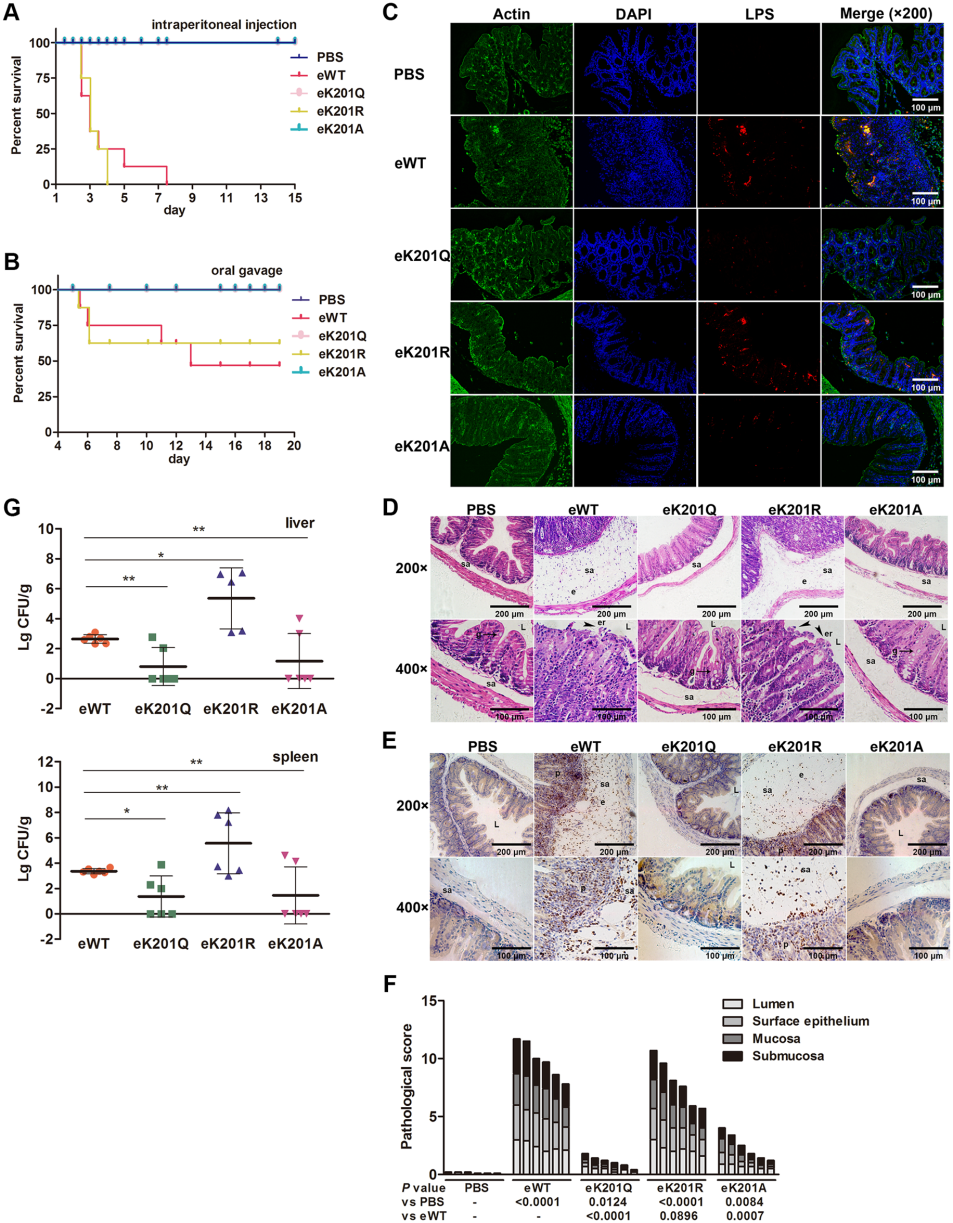
Acetylation of PhoP K201 modulates bacterial virulence in mouse model. (A) Survival rates of mice infected by intraperitoneal injection. 8-week-old BALB/c mice were administered intraperitoneally by 1.5 × 10^5^ bacteria in 100 μl PBS. Control mice were given 200 μl PBS. The number of live mice was counted twice a day. (B) Survival rates of mice infected by oral gavage. Inbred 8-week-old BALB/c mice were pre-treated with 20 mg streptomycin per mouse 24 h before infection. 1.5 × 10^7^ bacteria in 200 μl PBS were administered by oral gavage. Control mice were given 200 μl PBS. The number of live mice was counted twice a day. (C) Bacterial burdens in ceca of streptomycin-treated BALB/c mice. Paraformaldehyde-fixed paraffin sections were stained for actin (green), nuclei with DAPI (4’, 6’-diamidino-2-phenylindole; blue), or *Salmonella* LPS (red). Images are pseudocolor representations at ×200 magnification. For bacterial enumeration and histopathology, mice were euthanized by cervical dislocation at designated time points, and tissues were harvested aseptically. L, intestinal lumen; e, edema; er, erosion of the epithelial layer; sa, submucosa. Magnifications are indicated by the black bars. (D) H&E-stained ceca of streptomycin-treated BALB/c mice. Intestines of four groups of mice were fixed and embedded in paraffin, and then 5 μm thin sections were stained with H&E. (E) Neutrophil infiltration in ceca. Blue indicates the nucleus, which was stained by hematoxylin. Claybank indicates PMN. Sections were incubated with the anti-MPO antibody, followed by incubation with the anti-rabbit IgG HRP-linked antibody. Diaminobezidin (DAB) was used as chromogenic agent. (F) Pathological scores of ceca from PBS-, eWT-, eK201Q-, eK201R-and eK201A-infected mice at 48 h post-infection. Each bar represents a single mouse. To quantify pathological changes, H&E-stained sections of the ceca of six mice from each group were scored. Statistical analyses are shown for the separate scores and for the combined score. *P*, P value, statistical analysis was performed by using the exact Mann-Whitney U test and the SPSS version 19.0 software. (G) Bacterial burdens in liver and spleen. The livers and spleens were harvested 48 h after oral infection, and c.f.u. counting was performed to analyze the number of bacteria in these organs.

To determine the contribution of PhoP K201 acetylation to *S*. Typhimurium virulence in mouse model, eK201 mutants along with eWT were used to infect mice. Groups of 8 BALB/c mice were infected by intraperitoneal injection with 1.5 × 10^5^ eWT, eK201 mutants or Phosphate-buffered saline (PBS), and monitored for survival over a 15-day period. Mice infected with eWT or eK201R began dying on day 3, and eK201R-infected mice were all dead at day 4, which was 3.5 days earlier than eWT-infected mice (Fig 7A). However, mice inoculated with eK201Q or eK201A were alive and showed no morbidity during the whole experimental period. Therefore, eK201R-infected mice displayed shortest survival time, suggesting that non-acetylated PhoP guarantees the virulence of *S*. Typhimurium.

Next, we performed the survival assay by intragastric infection, and monitored mice survival over a 20-day period. Mice infected witheK201Q or eK201A were healthy and active throughout as PBS group mice while mice infected with eWT or eK201R appeared ruffled fur immediately after administration and began dying both at 5.5 days post-infection. At last, 5 eK201R- and 4 eWT-infected mice were alive and showed no infection-associated morbidity such as ruffling of fur and wasting (Fig 7B).

In addition, we studied bacterial colonization and the histopathologic features of mice cecal inflammation. Immunohistochemistry showed eK201Q and eK201A colonized less in mice ceca than eWT or eK201R (Fig 7C). Pathological changes including marked edema in submucosa, mast cell recruitment and loss of goblet cells occurred after 48 h infection with WT or eK201R, but absent from eK201Q- or eK201A-infected mice (Table 1). Moreover, desquamation in the surface epithelium layer was obvious in eWT- or eK201R-infected mice ceca (Fig 7D). We also observed the polymorphonuclear neutrophil (PMN) infiltration of the submucosa, the lamina propria, and the epithelial layer, as well as transmigration of PMN into the intestinal lumen (Fig 7E). Histopathologic scoring scheme for cecal tissue from six mice were shown in Fig 7F, indicating that *S*. Typhimurium-elicited ceca inflammation is more pronounced in eWT- or eK201R-infected mice, in line with the number of bacterial burdens in mice ceca, while no severe inflammations were observed in eK201Q- or eK201A-infected mice. The livers and spleens were harvested at 48 h post-infection, and c.f.u. counting was performed to analyze the number of bacteria in these organs. Consistently, infection of eK201Q or eK201A resulted in significant less bacterial burdens in both spleen and liver compared to eK201R and eWT strains (Fig 7G). Additionally, we observed higher burdens of eK201R than eWT in these organs, suggesting that the non-acetylated of PhoP is beneficial to *S*. Typhimurium colonization in host.

**Table 1:** Inflammatory pathology in the ceca of PhoP eWT, eK201Q, eK201R, and eK201A mutants infected mice.

Pathological change	Strains^a^					*P* value (ANOVA)^b^
	PBS	eWT	eK201Q	eK201R	eK201A	
Submucosal edema (% wall thickness)	18±5	38±11^1^ **	21±6^2^ **	37±14^1^ **	24±6^2^ *	<0.0001
Mucosa (μm)	105±17	301±67^1^ **	113±21^2^ **	279±74^1^ **	177±29^1^ * ^2^ *	<0.0001
Submucosa (μm)	24±14	184±87^1^ **	30±19^2^ **	171±69^1^ **	59±27^2^ **	0.0003
Goblet cells/HPF^c^	32±5	7.2±4^1^ **	26±6^2^ **	6.3±3^1^ **	21±6^2^ **	<0.0001
Mast cells/100 crypts	0.5±0.3	15±5.9^1^ **	1.0±0.6^2^ **	15±6.2^1^ **	4.5±1.8^1^ ** ^2^ **	0.0009

^*a*^ Values shown represent means ± standard deviation for each group.

*, *P*<0.05;

**, *P*<0.01;

by Tukey’s multiple comparison test;

^1^, versus PBS;

^2^, versus eWT

^*b*^ ANOVA, analysis of variance

^*c*^ HPF, high-powered field

## Discussion

### Acetylation of PhoP K201 is critical for regulation of PhoP activity

In this study, our results revealed that a conserved response regulator in bacterial, PhoP could be enzymically acetylated by acetyltransferase Pat (Fig 1C) and deacetylated by NAD-dependent deacetylase CobB *in vitro* (Fig 1B). The gene *pat* was involved in regulating the levels of *phoP* transcription both *in vitro* and *in vivo* (Fig 2A and 2C). With sequence alignment, we found PhoP K201 of *S*. Typhimurium was conserved and homologous to PhoP K224 of *M*. *tuberculosis* (Fig 3A). As reported, the C-terminal domain of PhoP exhibits a winged helix–turn–helix motif, and the molecular surfaces around the recognition helix (α8) display strong positive electrostatic potential, which indicates its role in the DNA-binding and nucleotide sequence recognition in *M*. *tuberculosis* [50].Therefore, mutations in this region affect DNA-binding ability of PhoP [31, 50]. Mass spectrometry showed K201 was acetylated *in vivo* (S3 Fig) and EMSA revealed that K201 was indeed an essential residue for DNA-binding ability of PhoP in *S*. Typhimurium (Fig 3B). K201Q avoids deacetylation but neutralizes positive charge can be thought of as mimicking the acetylation status of PhoP since it is functionally similar to acetylation [51]. K-to-Q mutant behaved like the K-to-A mutant (Fig 3B) and PhoP K201Ac also showed a decreased binding ability to *phoP* promoter (Fig 4D). Therefore, we consider that acetylated K201 appears to be critical for DNA-binding activity of PhoP. We further observed the decrease of acetylation levels of PhoP K201 in different physiological conditions, including low concentration of Mg^2+^, acid stimulation and phagocytosis by macrophages (Figs 5A, 5E and 6E). These results highlighted that the acetylation level of lysine residue in the DNA-binding domain affects the function of PhoP as a transcriptional regulator. Afterwards, ChIP assay validated that PhoP had an enhanced affinity to its target genes’ promoters in the *pat* deletion mutant (Fig 4F), and the constitutive acetylation of K201 (mimicked by K201Q) significantly impaired its DNA-binding ability *in vivo* (Figs 5D and 6C). Considering the location of PhoP K201, it is likely that acetylation on this residue disrupts the direct interactions between PhoP and PhoP box sequence.

In terms of the positive charge of PhoP K201, the molecular mechanism behind the observed attenuated DNA-binding ability that resulted from acetylation at K201 is probably due to neutralization of its positive charge, which would disrupt or impede the direct interactions with the negatively charged phosphate backbone of DNA in PhoP box. There are other reports about transcription factors whose DNA-binding activity was attenuated by acetylation, including RcsB controlling cell division, capsule and flagellum biosynthesis [24], and Foxo1, a member of the FOXO family of fork head transcription factors in eukaryote [52]. Therefore, we conclude that, as one of post-translation modification, reversible N^ε^-Lys acetylation of transcription factors is a conserved and elaborate style to regulate gene expression in both eukaryotes and bacteria.

It has been shown that PhoP phosphorylation is required for activation of transcription of *phoP* and PhoP-regulated genes [7]. We determined that the decreased DNA-binding ability of PhoP caused by acetylation of K201 was not due to D52 phosphorylation defect, because PhoP could be phosphorylated in both acetylated and non-acetylated statuses (Fig 4G). In fact, phosphorylation was still required for PhoP activity even on the PhoP K201R background (Fig 6D). These results again support that phosphorylation is indispensible for PhoP activity *in vivo*, and further demonstrate that the acetylation of K201 directly inhibits PhoP binding to its target genes’ promoters and does not affect PhoP phosphorylation. It is not clear whether phosphorylation blocks acetylation of PhoP K201 or promotes deacetylation of PhoP K201 to increase PhoP DNA-binding ability, this issue needs to be investigated further.

### Acetylation coordinates PhoP activity to control the expression of SPIs

As we know, *phoP* has two promoters in *S*. Typhimurium, the autoregulated P1 promoter and the constitutive P2 promoter. Shin *et al*. showed that the loss of the autoregulated P1 promoter of *phoP* attenuated the virulence of *S*. Typhimurium in mice, demonstrating that the activation of *phoP* was required for virulence [53]. Consistent with this finding, we showed that acetylation of PhoP K201 blocked PhoP from binding to its target DNA sequences, including the autoregulated P1 promoter. Both results were involved in regulating PhoP function by inhibiting the activity of P1 promoter. Moreover, K201Q mutant mimicking acetylated form of PhoP attenuated in mice, while K201R mutant mimicking the non-acetylated form of PhoP possessed a better replication ability in macrophages (Fig 6A and 6B), higher virulence in mouse model (Fig 7A) and more bacterial burdens in organs compared with the wild type strain (Fig 7G). All of them suggest that K201R can properly mimic the activated form of PhoP and function well in intracellular bacterial replication and systemic infection. The underlying reason is that PhoP K201R kept the ability to activate *phoP* transcription initiated from P1 promoter and PhoP-regulated gene transcription during infection, while PhoP K201Q inhibited these genes transcription due to its low DNA-binding affinity.

A series of regulatory systems have been identified that control the proper expression of virulence factors in a precise spatiotemporal manner, and the PhoP-PhoQ regulatory system is pivotal in this process [40]. PhoP upregulates the expression of SPI-2 by directly activating transcription of *ssrB*, therefore PhoP is indispensible for *Salmonella* within phagosomes [46]. In contrast, it has been established that PhoP suppresses bacterial invasion by downregulating the transcription of the SPI-1 master regulator, HilA. Therefore, repression of the PhoP-PhoQ is required during the early invasion steps [54]. The qPCR result showed that expression of SPI-1 genes including *sipC*, *prgI*, *invF*, *spaS* and two regulator genes *hilA* and *hilD* was significantly upregulated in eK201Q mimicking acetylation (S8 Fig). These results demonstrate that non-acetylated K201 representative of the activated PhoP suppresses the expression of SPI-1, while acetylated K201 representative of the inactivated PhoP is beneficial for the function of SPI-1 and bacterial invasion.

### Other acetylation factors might be involved in the acetylation of PhoP

We here demonstrated that the Gcn5-like acetyltransferase Pat played a major role in the acetylation of PhoP K201 in *S*. Typhimurium. However, Western blot showed that PhoP proteins from the *pat* deletion mutants still had acetylation signals (Figs 1A and 4A). It means Pat is responsible for part of PhoP acetylation but not all. The residual acetylation may be Pat-independent. Since AcP is a critical factor for protein acetylation in a non-enzymatic catalysis manner in *E*. *coli* [16–18], *S*. Typhimurium may utilize AcP to acetylate other lysine residues in PhoP. In addition to non-enzymatic AcP-dependent acetylation, there are possibilities that non-enzymatic Ac-CoA-dependent acetylation as well as other currently unidentified lysine acetyltransferases might exist [14]. Also the observation that some acetylation was CobB-insensitive (Fig 1B) as evidence that some acetyllysine is not deacetylated by CobB [18]. PhoP and particularly K201 are hyperacetylated in the *cobB* mutant (Fig 4A). We also show that acetylation of K201 decreases DNA-binding (Fig 3B). But the hyperacetylated PhoP in the *cobB* mutant did not affect phoP transcription (S2 Fig). We speculate that other unknown deacetylases, acetyltransferases or other factors might play a role by regulating *phoP* transcription *via* some unknown lysine residues. For example, YcgC was recently found to catalyze lysine deacetylation in *E*. *coli* [55]. This novel deacetylase does not share amino acid sequence homology to any known deacetylases and presents a new family of prokaryotic deacetylase. It is highly possible that other factors may be responsible for the acetylation of PhoP at residues other than K201. Actually we have already identified such modification at K18 and K150 by mass spectrometry. We presume more acetylated lysine residues may be detected by changing bacterial growth conditions, or with more sensitive mass spectrometry technology in the future.

The ATR enables *S*. Typhimurium to survive in exposure to lethal acidic environment. When accessed to inorganic acid stress, PhoP-PhoQ is activated to produce a set of acid shock proteins [10]. Besides, low Mg^2+^ level is also important in activation of this system [7]. Therefore, the environmental cue within the macrophage that triggers the PhoP-PhoQ cascade remains undetermined but probably reflects a combination of low level of Mg^2+^ and high level of H^+^. IP and ChIP assays showed that PhoP K201 was in a low acetylation state in macrophage (Fig 6E) and log-phase ATR (Fig 5E), which was critical for PhoP to activate its target genes. These findings demonstrate how the protein acetylation status responds to relevant stimuli and provided evidence that it is important for pathogenesis in *S*. Typhimurium. In conclusion, we propose that the regulation of transcriptional factors by acetylation of DNA-binding domain might be a conserved and universal mechanism to control gene expression under different environments.

## Materials and Methods

### Bacterial strains, plasmids, primers and media

All strains, plasmids and primers used in this study were described in Supplementary S1 and S2 Tables. Luria-Bertani broth (LB) was used as a rich medium, nutrient agar plates contained 1.5% (W/V) agar and supplemented with antibiotics as required. The antibiotics used were 100 μg/ml of ampicillin, 17 μg/ml of chloramphenicol, 50 μg/ml of kanamycin and 50 μg /ml of spectinomycin.

### Generation of chromosome knock-in strains

Fusion PCR and λ Red recombination were employed to construct *S*. Typhimurium PhoP K201Q, K201R, K201A and the wild type strain with or without Flag tag sequence at the C-terminus of *phoP*. The chloramphenicol resistance cassette was inserted between *phoP* and *phoQ*. PCR products were gel extracted and electroporated into *S*. Typhimurium containing pKD46 prepared in the presence of 10mM arabinose. The knock-in strains were verified by PCR and sequencing.

### Purification of PhoP and its derivative mutants

For purification of PhoP, pET22b*-phoP*, pQE80-*phoP*, pQE80-*phoP* K201Q, pQE80-*phoP* K201R and pQE80-*phoP* K201A were constructed and verified by sequencing. All the constructed plasmids were introduced individually into *E*. *coli* strain BL21, and the resultant strains were grown in LB medium containing 100 μg/ml ampicillin at 37°C. IPTG was added to a final concentration of 0.5 mM at the time point when the absorbance of the culture at 600 nm reached 0.6–0.8. The culture was continuously incubated for 5 h at 25°C. Afterwards cells were harvested by centrifugation, and stored at -80°C.

All subsequent procedures were performed at 4°C. Thawed bacteria were resuspended in lysis buffer (50 mM Tris–HCl, 0.6 M NaCl, 10% (vol/vol) glycerol, pH 7.5), supplemented with 20 mM DNase I and 0.1 mg/ml phenylmethylsulfonyl-fluoride (PMSF), then were broken by high pressure cracker. Any insoluble material was removed by centrifugation for 60 min at 19,000g. The soluble extract was applied to a 1 ml column of nickel-nitrilotriacetic acid (Ni-NTA) agarose (Qiagen) that had been equilibrated with lysis buffer containing 0.1 mg/ml PMSF. The column was subsequently washed with 10 ml of buffer A (50 mM HEPES, 0.2 M NaCl, 10% (vol/vol) glycerol, pH 7.3) supplemented with 1.2 M NaCl (final concentration), 5 ml of buffer A plus 15 mM imidazole, respectively, and eluted stepwise with 2 ml aliquots of buffer A containing 50 mM, 100 mM, 150 mM, 200 mM or 250 mM imidazole. For the protein whose purity is less than 90% was applied to a 1 ml HiTrap Q HP anion-exchange columns (GE Healthcare) as described previously [56]. The polypeptide compositions of the column fractions were monitored by 12% SDS–PAGE, and subsequent staining with Coomassie blue.

### Purification of Pat and CobB

Pat and CobB were overexpressed and purified by the similar procedure. BL21/pQE80-*pat* and BL21/pQE80-*cobB* were induced by final concentrations of 0.5 mM IPTG at OD_600_~0.8 for 4 h at 30°C. Cells were harvested by centrifugation, resuspended in Binding Buffer (20 mM Tris-HCl (pH 7.6), 500 mM NaCl and 20mM imidazole), and then disrupted using an ultrasonic processor (Sonics, Newtown, CT, USA). The disrupted suspension was centrifuged, and the resulting supernatant was loaded onto a 1 ml Ni-NTA column (GE Healthcare, USA). The column was washed initially with Washing Buffer (20 mM Tris-HCl (pH 7.6), 500 mM NaCl and 40 mM imidazole) and the histidine-tagged protein was eluted with Elution Buffer (40 mM Tris-HCl (pH 7.5), 500 mM NaCl and 500 mM imidazole). Protein purity was estimated to be > 90% on the basis of SDS-PAGE.

### *In vitro* acetylation assay

The *in vitro* acetylation reaction was performed in the buffer containing 50 mM Tris-HCl (pH 8.0), 0.1 mM EDTA, 10% glycerol, 1 mM dithiothreitol and 10 mM sodium butyrate. The acetylation was carried out by adding 10μgPat, 0.2 mM Ac-CoA, in a volume of 50 μl. Reaction mixtures were completely mixed and incubated at 37°C for 6 h. Specific protein concentrations are indicated in figure legends.

### *In vitro* deacetylation assay

Deacetylation of PhoP by CobB was performed in deacetylation reaction buffer including 50 mM Tris-HCl (pH 8.0), 135 mM NaCl, 2.5 mM KCl, 1 mM MgCl_2_ in the presence or absence of 1 mM NAD^+^, and in the presence or absence of 10 mM NAM. Specific protein concentrations and reaction times are indicated in figure legends.

### Site-directed mutagenesis of *phoP*

Site-directed mutagenesis of *phoP* was performed with the corresponding primers (S2 Table), using KOD-Plus-Mutagenesis Kit according to the manufacturer's recommendations (Toyobo, SMK-101). The resulting gene mutations were confirmed by DNA sequencing.

### Bacterial two-hybrid assay

The protein-protein interaction between PhoP and Pat or CobB were detected by Bacterial Two-Hybrid System (Stratagene) according to the previous procedure [57]. The *phoP* and *pat* or *cobB* genes were cloned into pBT and pTRG, respectively. The reporter strain was co-transformed with the recombinant vectors pBT-*phoP* and pTRG-*pat* or pTRG-*cobB*, and then spotted onto screening medium containing 8 mM 3-amino-1, 2, 4-triazole (3-AT), 12 mg/ml streptomycin, 15 mg/ml tetracycline, 34 mg/ml chloramphenicol and 50 mg/ml kanamycin. A co-transformant containing pBT-LGF2 and pTRG-Gal11Pwas used as a positive control for expected growth on the screening medium. A co-transformant containing empty vector pBT and pTRG was used as a negative control.

### Identification of acetylated lysine residues by mass spectrometry

The purified PhoP proteins were separated on 12% SDS-PAGE. The excised bands containing PhoP were destained and dehydrated. For trypsin digestion, proteins were treated with 100 mM DTT at 56°C for 30 min, and then mixed with 100 mM NH_4_HCO_3_ for 15 min at 25°C. The freeze-dried samples were incubated with 100–200 ng trypsin at 37°C for 20 h. Peptides were separated by the EASY-nLC HPLC system (Thermo Scientific, USA) and analyzed by Q-Exactive mass spectrometer (Thermo Scientific, USA). Mass spectrometric data were analyzed using the Mascot 2.2 software for database search.

### Primer extension

The primer extension assay was carried out according to the protocol previously developed by Fekete *et al* [58]. Specifically, 50 μg total RNA was dissolved in PIPES buffer (80 mM PIPES pH 6.4, 400 mM NaCl, 1mM EDTA) and heated at 65°C before5’-FAM-labeled primer PE-*phoP*-R was added. The reaction system was then slowly cooled to room temperature. A final concentration of 0.3 M NaAc and 2 volumes of anhydrous ethanol were added to precipitate the nucleic acids, which were subsequently washed twice with 70% ethanol, and finally dissolved in RNase-free water. Primer extension assay was performed at 50°C for 1 h in a 100μl reaction system, containing 1×first-strand buffer (Thermo), 1mM dNTPs, 2 μl RNase Inhibitor (TaKaRa) and 2 μl SuperScript III reverse transcriptase (Thermo). Reaction was stopped by heating inactivation at 95°C for 5 min, and 1 μg RNase A was added to remove the total RNA. cDNA was purified with the Wizard SV Gel and PCR Clean-Up System (Promega). The cDNA sample was then added with HiDi formamide (Applied Biosystems) andGeneScan-LIZ500 size standards (Applied Biosystems) before being analyzed with 3130xl DNA analyzer and Peak Scanner software v1.0 (Applied Biosystems).

### Electrophoretic mobility shift assay (EMSA)

DNA fragments used for EMSA were amplified by PCR using *Salmonella enterica* serovar Typhimurium genomic DNA as a template. The promoter region of *phoP-phoQ* was amplified using primers phoP-F (6’FAM-TCGCGCTGTGACTCTGGTCG) and phoP-R (6’FAM–ATCCTCTACAACCAGTACGC). PCR amplification rendered fragments of 184 bp. Approximately 1 nmol of 6’FAM-labeled DNA in a 10 μl volume was incubated at 37°C for 30 min with the indicated amounts of purified PhoP protein. The binding buffer used for protein-DNA incubations contained 25 mM Tris-HCl (pH 8.0), 50 mM KCl, 0.5 mM EDTA, and 10% glycerol. Samples were separated on a 5% non-denaturing Tris-glycine polyacrylamide gel at 4°C. FAM-labeled fluorescence was detected by the Fujifilm FLA7000.

### Surface plasmon resonance (SPR)

SPR measurements were performed on a Biacore 2000 using a streptavidin sensor chip (Biacore, GE). The protocol of SPR was described previously [33]. The PhoP box of *phoP* promoter was prepared by annealing a 5’-biotinylated oligonucleotide (biotin-GGGGTCTGGTTTATTAACTGTTTATCCGGGG) to a non-biotinylated complementary oligonucleotide (CCCCGGATAAACAGTTAATAAACCAGACCCC).

### Western blot

The standard Western blot procedure was described previously [59]. For acetylation Western blot, 50 mM Tris (pH 7.5) with 10% (vol/vol) Tween-20 and 1% peptone was used for blocking and 50 mM Tris (pH 7.5) with 0.1% peptone was used to prepare primary and secondary antibodies.

### Quantitative real-time PCR assay

Bacteria were cultured in EG medium at pH 7.7 to log phase, harvested and disrupted by Beads beater. RNA was isolated using Trizol (Invitrogen), and DNase I digestion was conducted as described previously [60]. Primers for qPCR were listed in S2 Table. Samples were run in triplicates and amplified using Real-time fluorescent quantitative reagents (TAKARA). The relative transcriptional level was determined by the methods of 2^−ΔΔCt^. 16S rRNA was used as a reference gene.

### PhoP polyclonal antibody preparation

The 6×His-tagged PhoP was overexpressed by using pET22b-*phoP* in *E*. *coli* strain BL21. Cells were induced with 0.5 mM isopropyl-β-D-thiogalactopyranoside (IPTG) and incubated for 5 h at 25°C. The 6×His-tagged PhoP was purified by nickel affinity chromatography and used to immunize rabbits for the production of polyclonal antibody.

### The anti-PhoP K201Ac specific polyclonal antibody preparation

The immune peptide GRLRKK(Ac)IQAQYPHD was used as antigen to immunize rabbits. During two months, rabbits were immunized for four times, and the antiserum was collected, and control peptide GRLRKKIQAQYPHD was used to remove non-specific antibody. The sensitivity and specificity of antibody were evaluated by ELISA and Western blot.

### Expression and purification of site-specifically acetylated PhoP (K201Ac)

*E*. *coli* strain BL21 was transformed with plasmid pAcKRS-3 and pCDF-PylT-*phoP* K201(TAG) and then were grown overnight in LB supplemented with 50 mg/ml kanamycin and 50 mg/ml spectinomycin (LB-KS). One liter of prewarmed LB-KS was inoculated with 20 ml overnight culture and was incubated at 37°C. When OD_600_ reached 1.8, the culture was supplemented with one liter of fresh LB with 20 mM acetyllysine (AcK). Protein expression was induced by addition of 0.5 mM IPTG for 6 h at 25°C, cells were harvested by centrifugation, and stored at -80°C. The purification method was described in “**Purification of PhoP and its derivative mutants**”.

### Protein concentration

Protein concentration was determined using the Bradford reagent with bovine serum albumin (BSA) as a standard [61].

### Log-phase acid tolerance response (ATR)

Log-phase ATR was measured as described previously [62]. Log-phase cells were obtained as follows. A 3.0 ml starter culture was inoculated with a single colony of the strain to be tested. The overnight culture (37°C, shaking) was diluted 1:100 in 5.0 ml of EG medium at pH 7.7. When the cultures reached a cell density of 2 × 10^8^ cells per ml (OD_600_ ~ 0.4), unadapted cells were challenged in EG medium at pH 3.3 (adjust the medium pH with HCl). Adapted cells were prepared by acid shock treatment in EG medium at pH 5.8 (adjust the medium pH with HCl) for 1 h before acid challenge. Viable-cell counts were determined for adapted and unadapted cells at 0 h, 1 h and 2 h post-acid challenge. Each experiment was performed three times. The data shown represent the average percentage of survival.

### Cell infection model

Mouse macrophage-like RAW264.7 cells obtained from Cell Resource Center of Shanghai Academy of Sciences, Chinese Academy of Sciences were seeded at 2×10^5^ per well in 24-well plates and grown at 37°C and 5% CO_2_ in DMEM supplemented with 10% fetal bovine serum (FBS), 100 units/ml penicillin G and 100 μg/ml streptomycin. The bacteria were diluted to achieve a multiplicity of infection (MOI) of 10, centrifuged at 400g for 5 min at 25°C to increase phagocytosis and incubated for 30 min at 37°C in 5% CO_2_. After extensive washing with DMEM twice, infected cells were incubated in fresh tissue culture medium containing 100 μg/ml gentamicin for the first 2 h post-infectionor15 μg/ml gentamicin for the remainder of the experiment. Infected cells were lysed at the desired post-infection time points with 0.1% (vol/vol) Triton X-100 in phosphate-buffered saline (PBS). The number of viable intracellular bacteria was determined by serial dilutions and plating. Bacterial growth was measured as the fold change in c.f.u. (colony-forming unit)/ml recovered from macrophages between two time points [63].

Thioglycolate-elicited primary peritoneal macrophages (Peritoneal MΦ) were harvested as described before [64]. Briefly, BALB/c mice were intraperitoneally injected with 4% Brewer’s thioglycolate medium (Sigma). After 3 days, mice were sacrificed by cervical dislocation, and cells were isolated by flushing the peritoneal cavity with 50 ml PBS per mouse. Cells were seeded in 24-well dishes, and non-adherent cells were removed by extensive washing with DMEM. The adherent peritoneal macrophages were used for subsequent experiments.

### Ethics statement

The animal procedures were approved by Shanghai Jiao Tong University School of Medicine, and this study was carried out in strict accordance with the National Research Council Guide for Care and Use of Laboratory Animals [SYXK (Shanghai 2007–0025)]. All surgery was performed under sodium pentobarbital anesthesia, and all efforts were made to minimize suffering.

### ChIP assay

ChIP of *Salmonella*-infected macrophages was performed as described previously [46]. RAW264.7 cells were seeded in 10 cm dishes with 1.7 × 10^7^ cells per dish and infected with bacteria containing a PhoP-Flag locus at MOI = 10for 1 h, washed three times and then incubated for 2 h containing 100 μg/ml gentamicin. Afterwards the cells were washed three times and incubated for another 22 h with 15μg/ml gentamicin. Subsequently, formaldehyde was added to each dish (final concentration 3%) and the plate was incubated at room temperature with occasional swirling. After 5 min, glycine at a final concentration of 125 mM was added to each well and the plate was incubated for another 5 min, washed twice with PBS and the cells containing the bacteria were harvested. The samples from ten dishes were pooled and used for ChIP following Chromatin Immunoprecipitation Kit (Millipore) protocol. The antibody for Chromatin immunoprecipitation (ChIP) was the anti-Flag antibody.

ChIP of *Salmonella* in acid stress was similar to the ChIP protocol in macrophages. Bacteria were cultured in EG medium at pH 7.7 to OD_600_ ~0.4, then centrifuged and resuspended in EG medium at pH 5.0 for another 1 h incubation. Afterwards, bacteria were harvested for ChIP assay.

### Immunoprecipitation

The antibody for immunoprecipitation (IP) was the anti-Flag antibody. The process was following Crosslink IP Kit protocol (Thermo). Finally, PhoP proteins immunoprecipitated were used for Western blot.

For bacteria containing a PhoP-Flag locus with different concentrations of magnesium treatment, bacteria were grown to OD_600_ ~0.4 in EG medium at pH 7.7 supplemented with 0 mM, 10 mM or 50 mM MgCl_2_, and then harvested for IP assay. As to bacteria containing a PhoP-Flag locus with mild acid treatment, bacteria were grown to OD_600_~0.4 in EG medium at pH 7.7 and treated with EG medium at pH 5.0 for 1 h, washed with PBS once, and then lysed in lysis buffer for IP. IP of *Salmonella*-infected macrophages performed was similar to the process of ChIP.

### Histological procedures

Ceca of experimental animals were fixed in 4% formalin overnight, followed by 18 h in 70% ethanol prior to being embedded in paraffin, sectioned, and stained with hematoxylin and eosin (H&E). As previously described, cecum pathology was evaluated by pathologists in a blinded manner and the following histopathological scoring scheme [65].

### Quantitative measurement of inflammation

Mast cells were counted at a magnification of ×400 from 100 sequential crypts. Goblet cells were enumerated spanning muscularis mucosa to surface epithelium with H&E-stained sections from 10 random high-powered fields. Submucosal thicknesses were measured at eight evenly spaced points per section for each experimental animal. Submucosa thickness was defined as the distance from muscularis mucosa to the muscularis externa. Averages for each mouse were compared.

### Immunohistochemistry staining

Paraffin-embedded tissues were deparaffinized as described above, then washed with PBS containing 0.1% bovine serum albumin (PBS-BSA). Sections were blocked in 10% goat serum in PBS-BSA at 25°C for 30 min. Sections were then washed three times in PBS-BSA, prior to incubation overnight at 4°C with primary antibody the anti-*Salmonella* lipopolysaccharide (2.5 μg/ml; LifeSpan Biosciences) and the anti-β-actin antibody (5 μg/ml; LifeSpan Biosciences). Sections then were washed three times in PBS-BSA, prior to incubation with the appropriate fluorochrome-conjugated secondary antibodies for 30 min at 25°C. Sections were incubated with 0.1 μg/ml DAPI at room temperature for 10 min shielded from light, then washed three times. Samples were observed using an inverted fluorescence microscope (Olympus), and a charge-coupled device (CCD) camera (Olympus) was mounted on the microscope. Fluorescent images were cropped and scaled by Adobe Photoshop CS6.

### Phosphorylation assay of PhoP

[^32^P]-AcP was synthesized with *E*. *coli* acetate kinase and [γ-^32^P]-ATP [66]. The reaction mixture contained 60 mM NaAc, 25 mM Tris-HCl (pH 7.4), 10 mM MgCl_2_, 0.5 U of acetate kinase (Sigma Aldrich) and 50 μCi of γATP (6000 Ci mmol^-1^, Perkin Elmer). The reaction was incubated at 25°C for 30 min and the AcP was separated from the enzyme using a Microcon-10 microconcentrator (Millipore). Four micrograms of PhoP WT, PhoP K201Ac and PhoP D52A proteins were separately incubated with [^32^P]-AcP for 30 min at 25°C. The reactions were stopped by addition of SDS sample buffer. Samples were subjected to SDS-PAGE, and gels were exposed overnight on a phosphor screen (GE Healthcare) followed by being visualized using the Fujifilm FLA7000 imager. Proteins were visualized by Coomassie brilliant blue staining.

### Accession numbers for genes/proteins mentioned in text

The gene ID numbers in Genbank: *pat* (ID: 11756548), *cobB* (ID: 11756861), *phoP* (ID: 11756864). *mgtA* (ID: 11756750), *mgtC* (ID: 11757770), *pmrA* (ID: 11754800), *pmrC* (ID: 11758088),*pmrD* (ID: 11753301) *pagC* (ID: 11755168), *pbgP* (ID: 11757346). The protein ID numbers in UniProt: Pat (A0A0F6B583), CobB (A0A0F6B059), PhoP (D0ZV90).

## Supporting Information

S1 Table Strains and plasmids used in this study. (XLSX)

S2 Table Primers used in this study. (XLSX)

S1 Fig Bacterial two-hybrid assays for the interaction between PhoP and Pat or CobB. The experiment was performed as described under “Materials and Methods” section. Left panel: plate minus streptomycin (str) and 3-amino-1, 2, 4-triazole (3-AT). Right panel: plate plus 12 mg/mL str and 8 mM 3-AT. CK^**+**^: co-transformant containing pBT-LGF2 and pTRG-Gal11P as a positive control. CK^**━**^: co-transformant containing pBT and pTRG as a negative control. Each unit represented the corresponding co-transformant in the plates was indicated in Figure.
(TIF)

S2 Fig The transcription of *phoP* in *pat* and *cobB* mutants. The transcriptional level was determined by qPCR with the methods of 2^−ΔΔCt^. The relative expression of *phoP* was normalized to that of 16S rRNA.
(TIF)

S3 Fig Representative mass spectrometry analysis of tryptic peptides derived from purified PhoP. 6×His-tagged PhoP was expressed using pCDSS-*phoP* and analyzed with LC/MS/MS after trypsin digestion. Shown is the spectrum covering the region from 200 to 1600 m/z which includes the peptide containing the acetylated lysine 201.
(TIF)

S4 Fig Purity of PhoP WT, PhoP K201Q, PhoP K201R and K201A. PhoP and its mutants were purified and desalted as described in “Materials and Methods”. 500 ng of PhoP, K201Q, K201R, and K201A were resolved on 12% SDS-PAGE respectively and stained with Coomassie bright blue.
(TIF)

S5 Fig Validation of influences of *phoP* chromosome genetic manipulations on bacterial growth and transcription of *phoP*-*phoQ*. We constructed PhoP K201Q, K201R, and K201A mutants at *phoP* locus in *S*. Typhimurium chromosome. After removing the chloramphenicol resistance cassette between *phoP* and *phoQ* locus, there is still a short DNA sequence residual in genome. PhoP eWT (engineered WT) has been performed the same gene manipulation as PhoP K201Q, K201R and K201A mutants. PhoP eWT served as the wild type strain when PhoP K201Q, K201R and K201A mutant stains were used. In order to confirm that the DNA sequence residual between *phoP* and *phoQ* locus does not affect bacteria growth and transcription of *phoP-phoQ*, we compared the *phoP-phoQ* mRNA levels (**A**) and growth rates (**B**) between WT and eWT. We also compared those characteristics between the wild type strain and *phoP* C-terminus Flag knock in strain (PhoP-Flag). The construction method was described in “Materials and Methods” section. For RNA isolation, the cells were grown to log phase (OD_600_~0.4) and harvested. (**C**) The transcriptional levels of *phoP* and *phoQ* were detected by qPCR. For (**D**) growth curve measurement, the strains were cultured overnight, diluted to OD_600_~0.1 with fresh LB medium, and the OD_600_ was recorded every hour.
(TIF)

S6 Fig Specificity of the anti-K201Ac antibody. Anti-K201Ac of PhoP polyclonal antibody was prepared as described in “Materials and Methods” section. (A) Anti-K201Ac antibody recognizes PhoP K201Ac, not PhoP K102Ac. (B) Anti-K201Ac antibody recognizes PhoP K201Ac and does not recognize PhoP K201Q, PhoP K201R and PhoP K201A.
(TIF)

S7 Fig Purity of wild type PhoP (WT) and PhoPK201Ac. The wild type PhoP (without site-specific acetylation) and PhoP K201Ac were purified and desalted as described in Materials and Methods. PhoP and PhoP K201Ac (500 ng) were resolved on 12% SDS-PAGE and stained with Coomassie bright blue.
(TIF)

S8 Fig The transcription of SPI-1 genes in chromosome *phoP* mutants. The chromosome *phoP* mutants were grown to OD_600_~0.4 in EG medium at pH7.7 and harvested to isolate total RNA. The transcriptional level was determined by qPCR with the methods of 2^−ΔΔCt^. The relative expression of tested genes was normalized to that of 16S rRNA.
(TIF)
