# Supplementary figures and images for: Acetylation of Lysine 201 Inhibits the DNA-Binding Ability of PhoP to Regulate Salmonella Virulence
Source: PLoS Pathog. 2016 Mar 4;12(3):e1005458. doi: 10.1371/journal.ppat.1005458 (PMC4778762; doi:10.1371/journal.ppat.1005458)

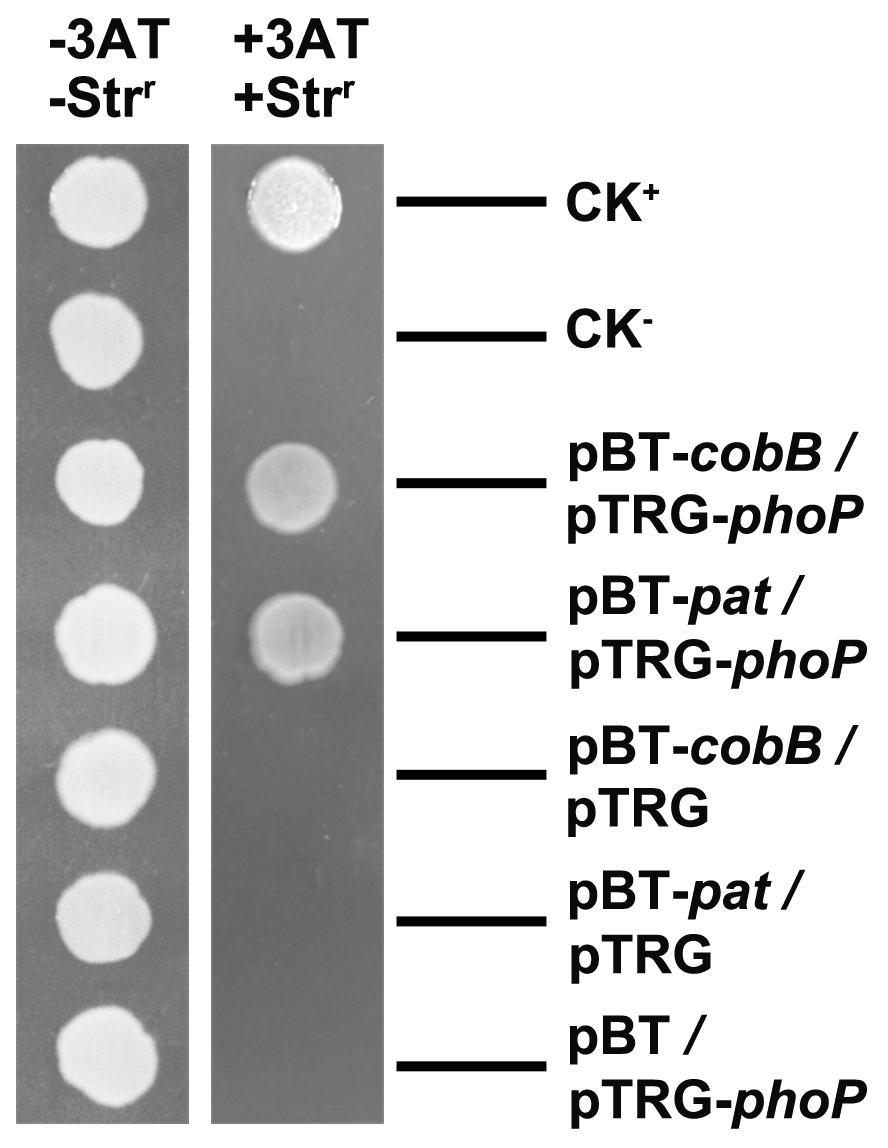

Supplement: S1 Fig — The experiment was performed as described under “Materials and Methods” section. Left panel: plate minus streptomycin (str) and 3-amino-1, 2, 4-triazole (3-AT). Right panel: plate plus 12 mg/mL str and 8 mM 3-AT. CK+: co-transformant containing pBT-LGF2 and pTRG-Gal11P as a positive control. CK━: co-transformant containing pBT and pTRG as a negative control. Each unit represented the corresponding co-transformant in the plates was indicated in Figure. (TIF) [file ppat.1005458.s003.tif]

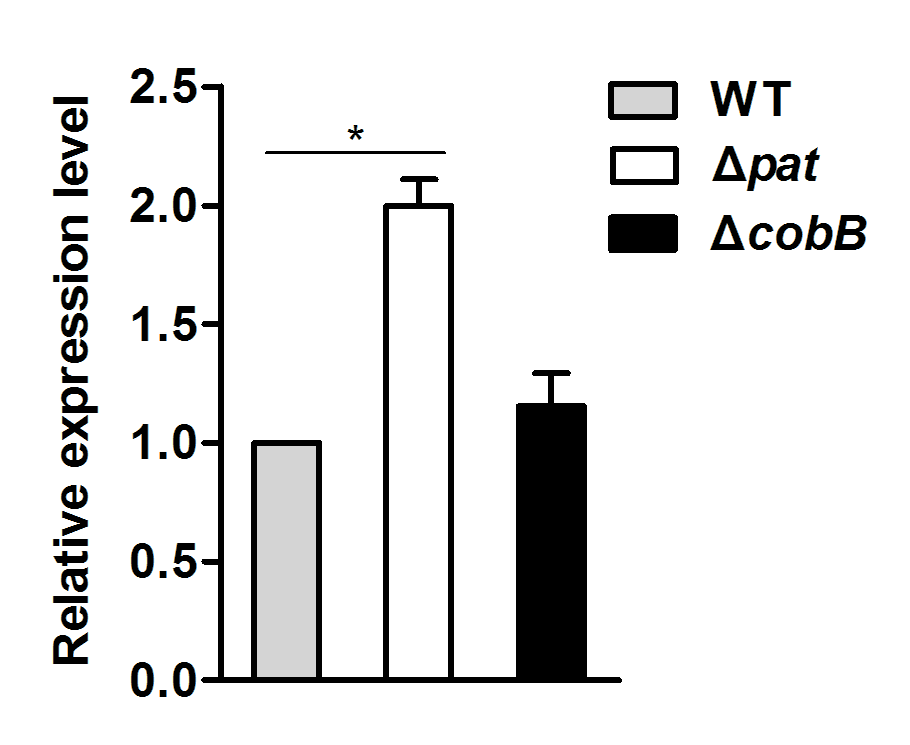

Supplement: S2 Fig — The transcriptional level was determined by qPCR with the methods of 2−ΔΔCt. The relative expression of phoP was normalized to that of 16S rRNA. (TIF) [file ppat.1005458.s004.tif]

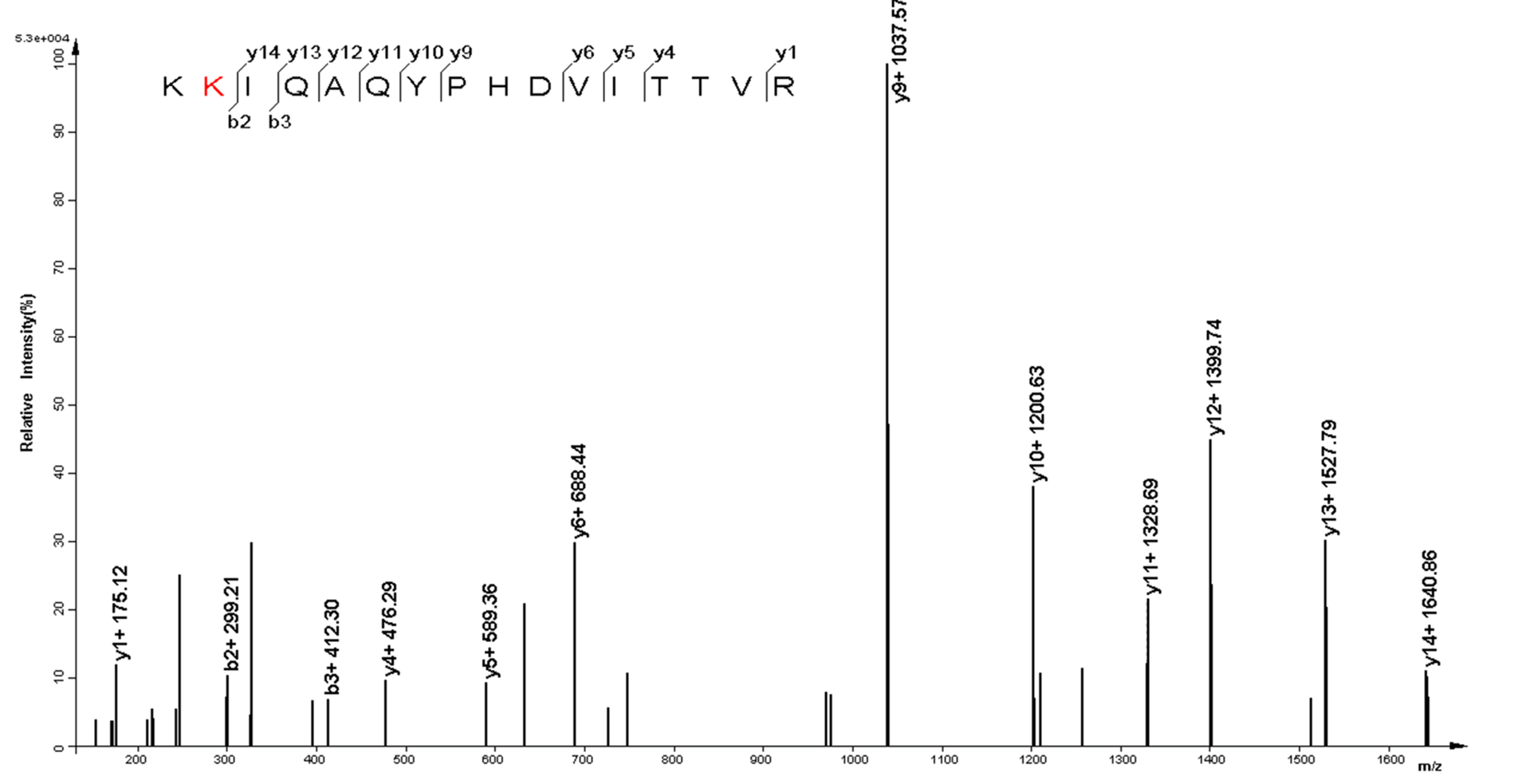

Supplement: S3 Fig — 6×His-tagged PhoP was expressed using pCDSS-phoP and analyzed with LC/MS/MS after trypsin digestion. Shown is the spectrum covering the region from 200 to 1600 m/z which includes the peptide containing the acetylated lysine 201. (TIF) [file ppat.1005458.s005.tif]

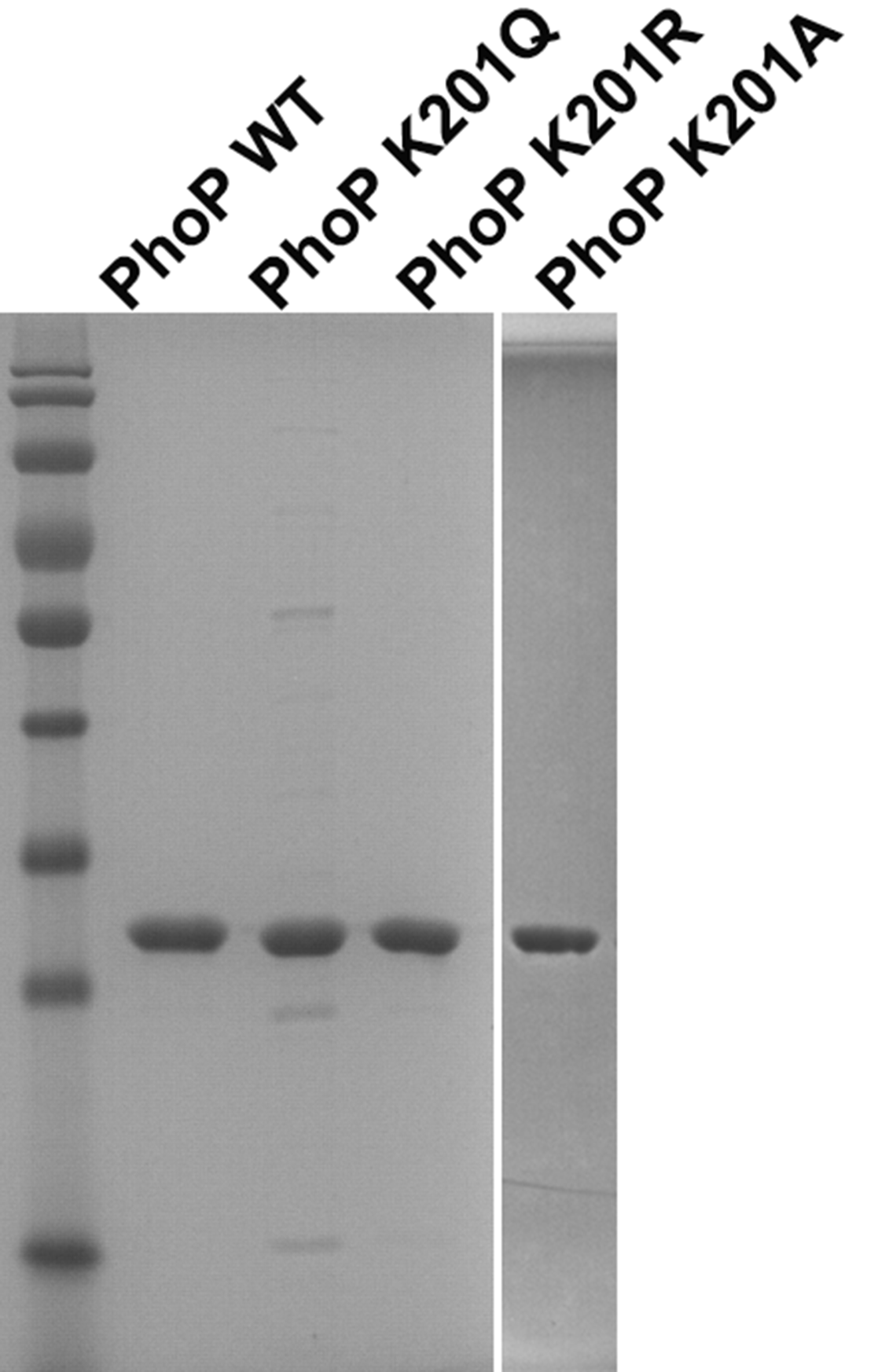

Supplement: S4 Fig — PhoP and its mutants were purified and desalted as described in “Materials and Methods”. 500 ng of PhoP, K201Q, K201R, and K201A were resolved on 12% SDS-PAGE respectively and stained with Coomassie bright blue. (TIF) [file ppat.1005458.s006.tif]

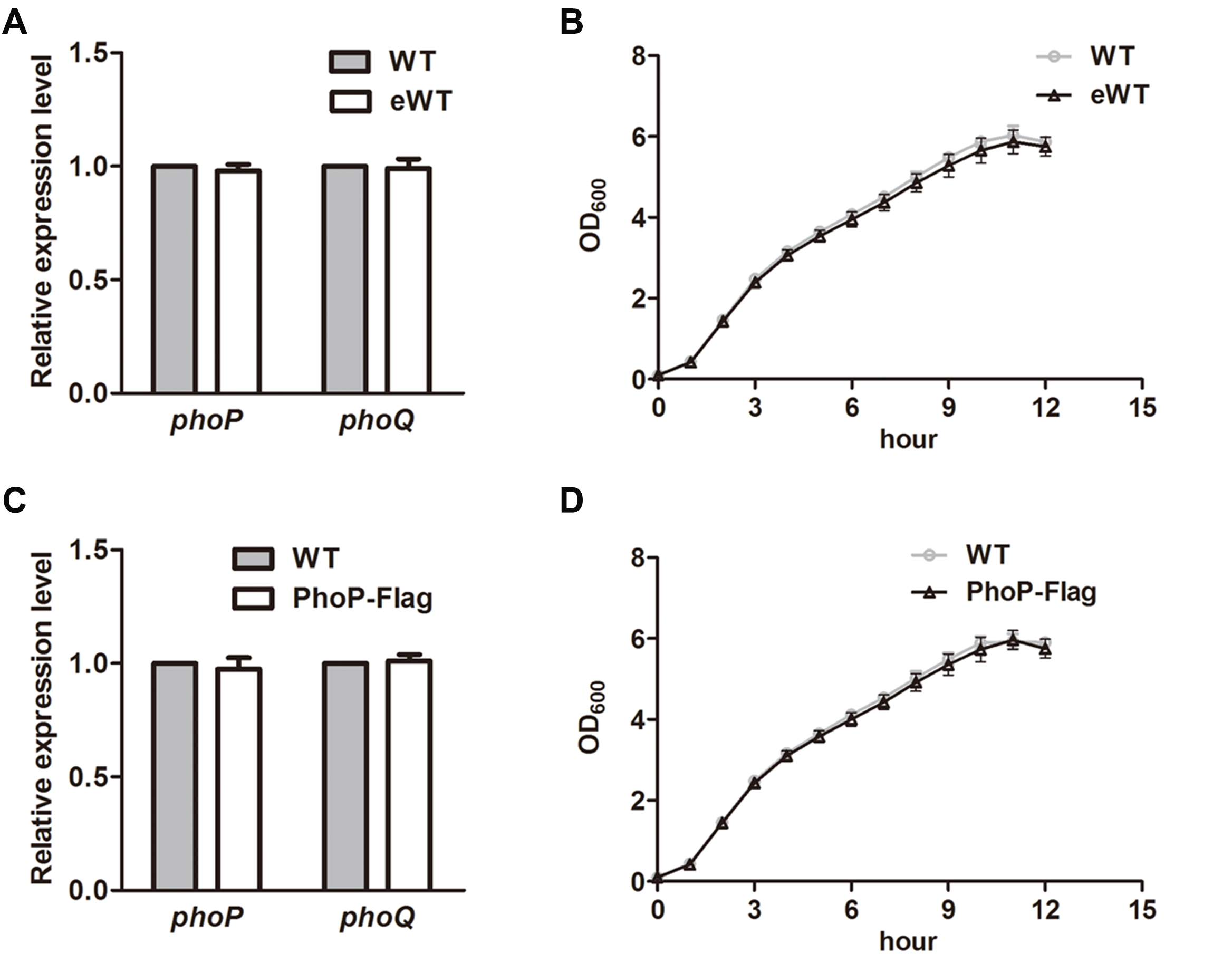

Supplement: S5 Fig — We constructed PhoP K201Q, K201R, and K201A mutants at phoP locus in S. Typhimurium chromosome. After removing the chloramphenicol resistance cassette between phoP and phoQ locus, there is still a short DNA sequence residual in genome. PhoP eWT (engineered WT) has been performed the same gene manipulation as PhoP K201Q, K201R and K201A mutants. PhoP eWT served as the wild type strain when PhoP K201Q, K201R and K201A mutant stains were used. In order to confirm that the DNA sequence residual between phoP and phoQ locus does not affect bacteria growth and transcription of phoP-phoQ, we compared the phoP-phoQ mRNA levels (A) and growth rates (B) between WT and eWT. We also compared those characteristics between the wild type strain and phoP C-terminus Flag knock in strain (PhoP-Flag). The construction method was described in “Materials and Methods” section. For RNA isolation, the cells were grown to log phase (OD600~0.4) and harvested. (C) The transcriptional levels of phoP and phoQ were detected by qPCR. For (D) growth curve measurement, the strains were cultured overnight, diluted to OD600~0.1 with fresh LB medium, and the OD600 was recorded every hour. (TIF) [file ppat.1005458.s007.tif]

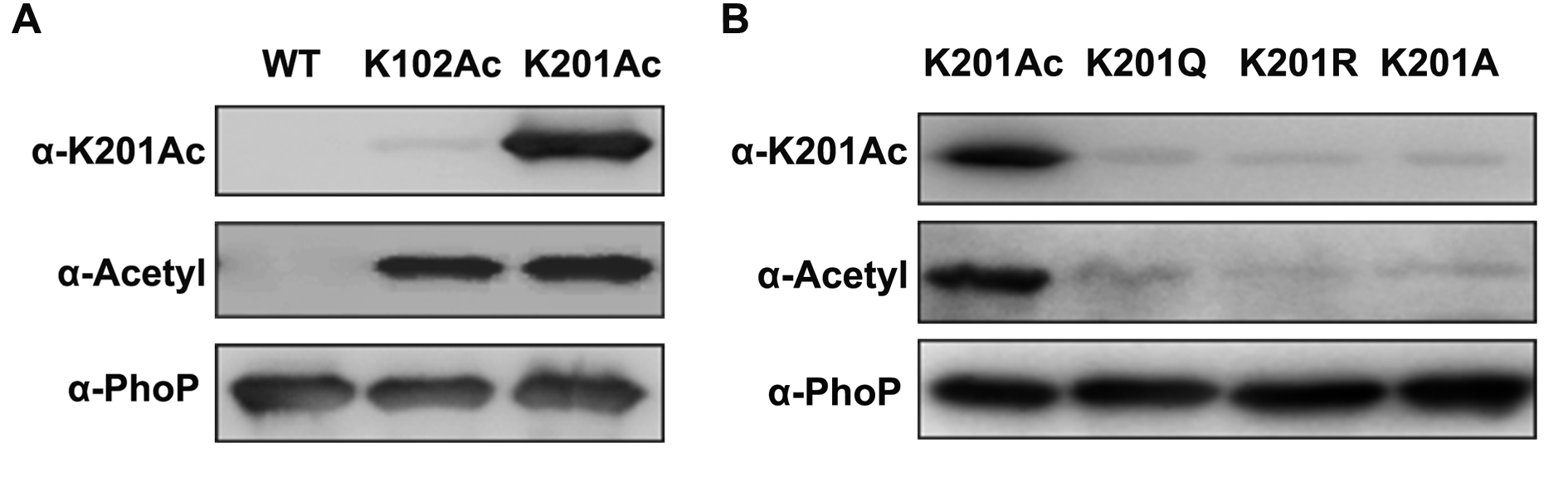

Supplement: S6 Fig — Anti-K201Ac of PhoP polyclonal antibody was prepared as described in “Materials and Methods” section. (A) Anti-K201Ac antibody recognizes PhoP K201Ac, not PhoP K102Ac. (B) Anti-K201Ac antibody recognizes PhoP K201Ac and does not recognize PhoP K201Q, PhoP K201R and PhoP K201A. (TIF) [file ppat.1005458.s008.tif]

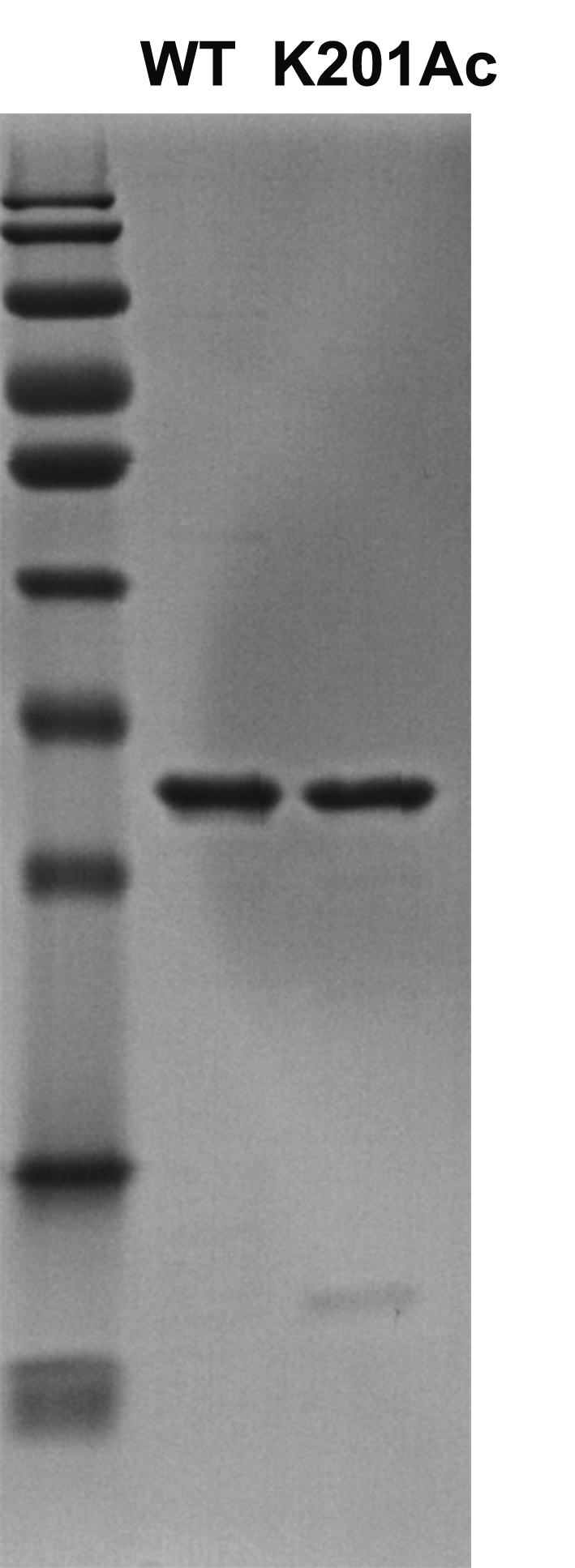

Supplement: S7 Fig — The wild type PhoP (without site-specific acetylation) and PhoP K201Ac were purified and desalted as described in Materials and Methods. PhoP and PhoP K201Ac (500 ng) were resolved on 12% SDS-PAGE and stained with Coomassie bright blue. (TIF) [file ppat.1005458.s009.tif]

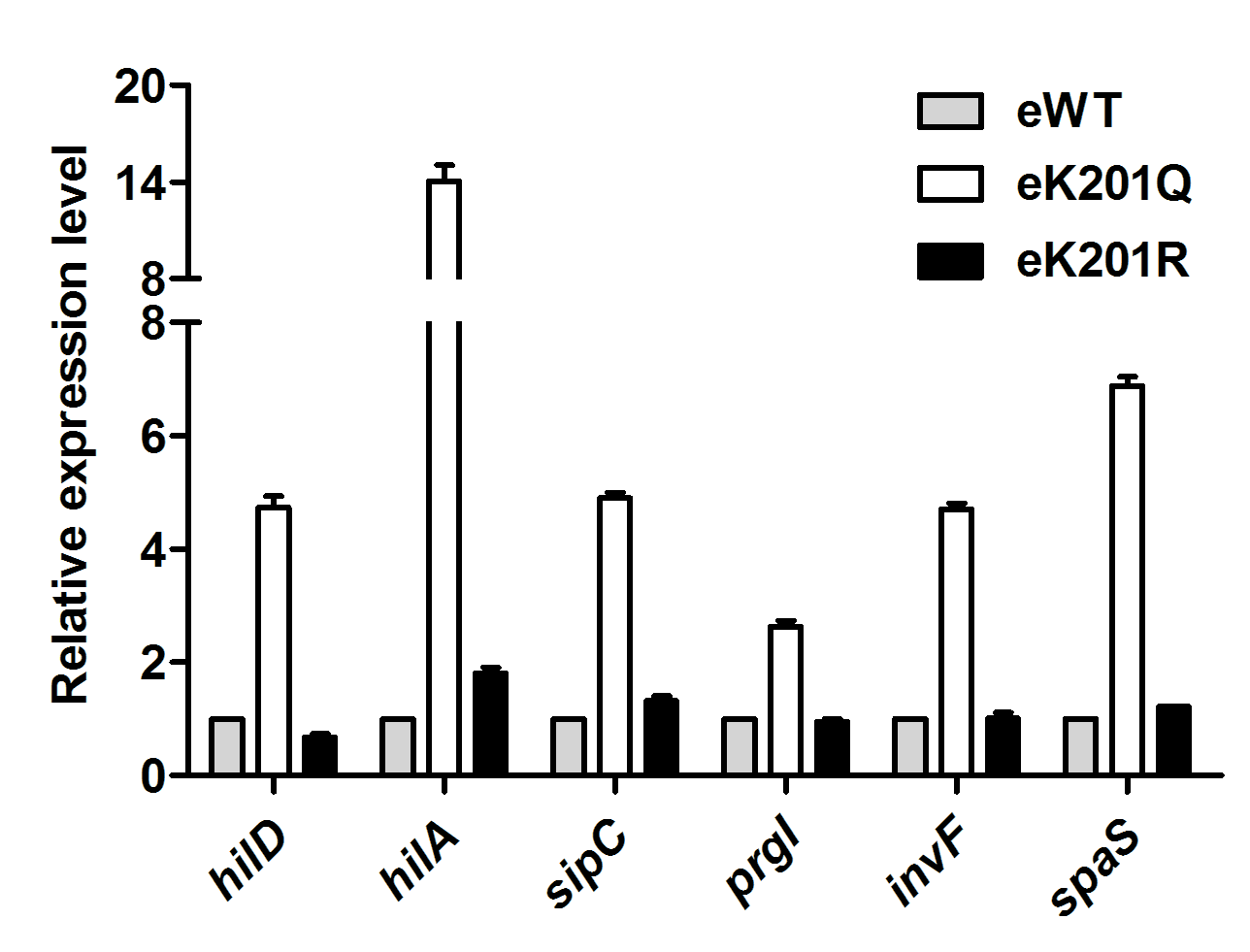

Supplement: S8 Fig — The chromosome phoP mutants were grown to OD600~0.4 in EG medium at pH7.7 and harvested to isolate total RNA. The transcriptional level was determined by qPCR with the methods of 2−ΔΔCt. The relative expression of tested genes was normalized to that of 16S rRNA. (TIF) [file ppat.1005458.s010.tif]
